# Association of Healthy Lifestyle With Years Lived Without Major Chronic Diseases

**DOI:** 10.1001/jamainternmed.2020.0618

**Published:** 2020-04-06

**Authors:** Solja T. Nyberg, Archana Singh-Manoux, Jaana Pentti, Ida E. H. Madsen, Severine Sabia, Lars Alfredsson, Jakob B. Bjorner, Marianne Borritz, Hermann Burr, Marcel Goldberg, Katriina Heikkilä, Markus Jokela, Anders Knutsson, Tea Lallukka, Joni V. Lindbohm, Martin L. Nielsen, Maria Nordin, Tuula Oksanen, Jan H. Pejtersen, Ossi Rahkonen, Reiner Rugulies, Martin J. Shipley, Pyry N. Sipilä, Sari Stenholm, Sakari Suominen, Jussi Vahtera, Marianna Virtanen, Hugo Westerlund, Marie Zins, Mark Hamer, G. David Batty, Mika Kivimäki

**Affiliations:** 1 Clinicum, Department of Public Health, Faculty of Medicine, University of Helsinki, Helsinki, Finland; 2 Department of Epidemiology and Public Health, University College London, London, United Kingdom; 3 Inserm U1153, Epidemiology of Ageing and Neurodegenrative Diseases, Paris, France; 4 Department of Public Health, University of Turku, Turku University Hospital, Turku, Finland; 5 Centre for Population Health Research, University of Turku, Turku University Hospital, Turku, Finland; 6 National Research Centre for the Working Environment, Copenhagen, Denmark; 7 Institute of Environmental Medicine, Karolinska Institutet, Stockholm, Sweden; 8 Centre for Occupational and Environmental Medicine, Stockholm County Council, Stockholm, Sweden; 9 Bispebjerg University Hospital, Copenhagen, Denmark; 10 Federal Institute for Occupational Safety and Health, Berlin, Germany; 11 Faculty of Medicine, Paris Descartes University, Paris, France; 12 Inserm UMS 011, Population-Based Epidemiological Cohorts Unit, Villejuif, France; 13 Department of Health Services Research and Policy, London School of Hygiene and Tropical Medicine, London, United Kingdom; 14 Department of Psychology and Logopedics, Faculty of Medicine, University of Helsinki, Helsinki, Finland; 15 Department of Health Sciences, Mid Sweden University, Sundsvall, Sweden; 16 Finnish Institute of Occupational Health, Helsinki, Finland; 17 AS3 Employment, AS3 Companies, Viby J, Denmark; 18 Stress Research Institute, Stockholm University, Stockholm, Sweden; 19 Department of Psychology, Umeå University, Umeå, Sweden; 20 VIVE–The Danish Center for Social Science Research, Copenhagen, Denmark; 21 Department of Public Health and Department of Psychology, University of Copenhagen, Copenhagen, Denmark; 22 University of Skövde, School of Health and Education, Skövde, Sweden; 23 School of Educational Sciences and Psychology, University of Eastern Finland, Joensuu, Finland; 24 Department of Clinical Neuroscience, Karolinska Institutet, Stockholm, Sweden; 25 Division of Surgery & Interventional Science, Faculty of Medical Sciences, University College London, London, United Kingdom; 26 School of Biological and Population Health Sciences, Oregon State University, Corvallis, Oregon

## Abstract

**Importance:**

It is well established that selected lifestyle factors are individually associated with lower risk of chronic diseases, but how combinations of these factors are associated with disease-free life-years is unknown.

**Objective:**

To estimate the association between healthy lifestyle and the number of disease-free life-years.

**Design, Setting, and Participants:**

A prospective multicohort study, including 12 European studies as part of the Individual-Participant-Data Meta-analysis in Working Populations Consortium, was performed. Participants included 116 043 people free of major noncommunicable disease at baseline from August 7, 1991, to May 31, 2006. Data analysis was conducted from May 22, 2018, to January 21, 2020.

**Exposures:**

Four baseline lifestyle factors (smoking, body mass index, physical activity, and alcohol consumption) were each allocated a score based on risk status: optimal (2 points), intermediate (1 point), or poor (0 points) resulting in an aggregated lifestyle score ranging from 0 (worst) to 8 (best). Sixteen lifestyle profiles were constructed from combinations of these risk factors.

**Main Outcomes and Measures:**

The number of years between ages 40 and 75 years without chronic disease, including type 2 diabetes, coronary heart disease, stroke, cancer, asthma, and chronic obstructive pulmonary disease.

**Results:**

Of the 116 043 people included in the analysis, the mean (SD) age was 43.7 (10.1) years and 70 911 were women (61.1%). During 1.45 million person-years at risk (mean follow-up, 12.5 years; range, 4.9-18.6 years), 17 383 participants developed at least 1 chronic disease. There was a linear association between overall healthy lifestyle score and the number of disease-free years, such that a 1-point improvement in the score was associated with an increase of 0.96 (95% CI, 0.83-1.08) disease-free years in men and 0.89 (95% CI, 0.75-1.02) years in women. Comparing the best lifestyle score with the worst lifestyle score was associated with 9.9 (95% CI 6.7-13.1) additional years without chronic diseases in men and 9.4 (95% CI 5.4-13.3) additional years in women (*P* < .001 for dose-response). All of the 4 lifestyle profiles that were associated with the highest number of disease-free years included a body-mass index less than 25 (calculated as weight in kilograms divided by height in meters squared) and at least 2 of the following factors: never smoking, physical activity, and moderate alcohol consumption. Participants with 1 of these lifestyle profiles reached age 70.3 (95% CI, 69.9-70.8) to 71.4 (95% CI, 70.9-72.0) years disease free depending on the profile and sex.

**Conclusions and Relevance:**

In this multicohort analysis, various healthy lifestyle profiles appeared to be associated with gains in life-years without major chronic diseases.

## Key Points

**Question:**

Are different combinations of lifestyle factors associated with years lived without chronic diseases?

**Findings:**

In a multicohort study of 116 043 participants, a statistically significant association between overall healthy lifestyle score and an increased number of disease-free life-years was noted. Of 16 different lifestyle profiles studied, the 4 that were associated with the greatest disease-free life years included body mass index lower than 25 and at least 2 of 3 factors: never smoking, physical activity, and moderate alcohol consumption.

**Meaning:**

Various healthy lifestyle profiles appear to be associated with extended gains in life lived without type 2 diabetes, cardiovascular and respiratory diseases, and cancer.

## Teaser

This cohort study examines disease-free life-years in participants with varying combinations of lifestyle risk factors.

## Introduction

Numerous observational studies over the past 80 years have explored the association of lifestyle risk factors, individually and, more recently, collectively, with the risk of mortality and chronic disease.^1,2^ Findings suggest that being physically active, being of normal weight, avoiding smoking, and consuming a moderate amount of alcohol confer the lowest risk of total mortality and chronic, noncommunicable disease, particularly cardiovascular disease. Uncertainty exists, however, with regard to the association of such a healthy lifestyle with life expectancy, particularly disease-free life expectancy, a measure that may be more useful for policy communication and public understanding than the ubiquitous relative risk estimates.^3,4,5,6^

The few existing investigations on disease-free life expectancy have reported mixed findings. A study of Dutch men and women found that those with all of the described healthy lifestyle factors lived 2 extra years in good health compared with those in the high-risk group,^3^ while in a multicohort analysis, those with no lifestyle risk factors lived an average of 6 years longer free of chronic diseases than those with at least 2 risk factors.^4^ In a further general-population sample, the absence of risk factors was associated with a 9-year delay in the mean age at onset of chronic diseases.^5^ While this body of evidence is informative, it remains unclear to what extent specific combinations of healthy lifestyle factors are associated with the number of years lived without major chronic disease.

The objective of this multicohort study therefore was to quantify the extent to which lifestyle factors in combination are associated with the number of disease-free life-years as indexed by the age at onset of the first major chronic disease. In these analyses, we focused on 16 lifestyle profiles based on combinations of 4 healthy lifestyle factors and 6 noncommunicable chronic diseases prioritized by the World Health Organization as targets for prevention (type 2 diabetes, coronary heart disease, stroke, cancer, asthma, and chronic obstructive pulmonary disease [COPD]),^7,8^ and expanded these diseases to include heart failure and dementia.

## Methods

### Study Population

This prospective multicohort study included 12 European cohorts from the Individual-Participant-Data Meta-analysis in Working Populations (IPD-Work) Consortium.^9^ Twelve of the 19 IPD-Work Consortium cohorts had data on all risk factors at baseline and follow-up of noncommunicable diseases and were included in this analysis (Figure): the United Kingdom (Whitehall II), France (Électricité de France-Gaz de France Employees, Denmark (Copenhagen Psychosocial Questionnaire study II, Danish Work Environment Cohort Study [2 cohorts], Intervention Project on Absence and Well-being, Burnout, Motivation, and Job Satisfaction), Finland (Finnish Public Sector, Health and Social Support, Helsinki Health Study), and Sweden (Work, Lipids, and Fibrinogen Stockholm; and Work, Lipids, and Fibrinogen Norrland). Participants were included in the analyses if they were free from the 6 chronic diseases at baseline and had information available on sex, age, socioeconomic status, lifestyle factors (weight, height, smoking, physical activity, and alcohol consumption), and follow-up for chronic diseases. Study baseline ranged from August 7, 1991, to May 31, 2006, and data analysis was conducted from May 22, 2018, to January 21, 2020. This study followed the Strengthening the Reporting of Observational Studies in Epidemiology (STROBE) reporting guideline for cohort studies.

**Figure.**
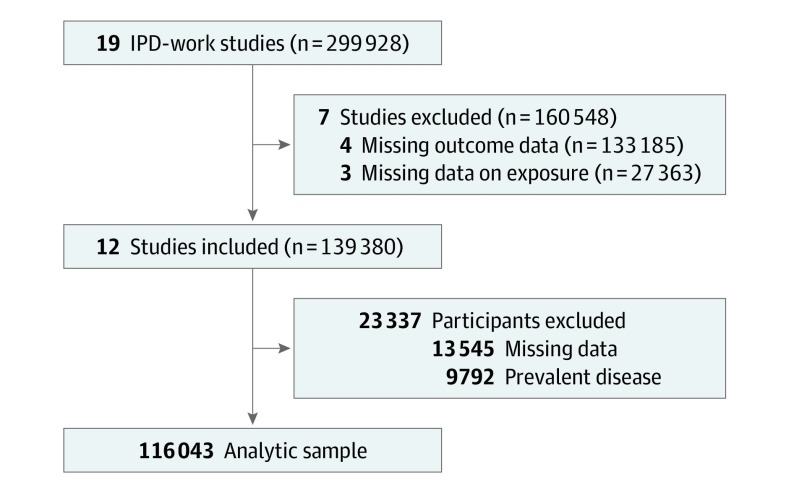
Flowchart of Sample Selection for Multicohort Analysis Derivation of the final analysis sample from the Individual-Participant-Data Meta-Analysis in Working Populations (IPD-Work) consortium. Twelve of the 19 IPD-Work Consortium cohorts that had data on 4 lifestyle factors at baseline and a follow-up of 6 chronic diseases (type 2 diabetes, coronary heart disease, stroke, cancer, asthma, and chronic obstructive pulmonary disease). Participants were included in the analyses if they did not have these diseases at baseline, had information on sex, age, socioeconomic status, lifestyle factors (weight, height, smoking, physical activity, and alcohol consumption), and had follow-up for these incident diseases.

All cohort studies in the IPD-Work Consortium received local ethical committee approval and written informed consent was obtained from study participants. To our knowledge, participants did not receive financial compensation. Details of study designs, participants and measurements are given in the eMethods in the Supplement.^10,11,12,13,14^

Lifestyle factors were body mass index (BMI) (calculated as weight in kilograms divided by height in meters squared), smoking, leisure-time physical activity, and alcohol consumption. The scoring system for each lifestyle factor was based on prespecified thresholds used in IPD-Work Consortium articles and was as follows:

- BMI: less than 25.0 (optimal), 25.0 to 29.9 (intermediate), and greater than or equal to 30.0 (poor).^15^
- Smoking: never smokers (optimal), former smokers (intermediate), and current smokers (poor).
- Leisure-time physical activity: Meeting the World Health Organization recommendations (≥2.5 hours of moderate activity/week or ≥1.25 hours of vigorous activity/week: optimal),^16^ activity levels falling between the optimal and poor levels (intermediate); and no or very little moderate/vigorous leisure-time physical activity (poor).^12^
- Alcohol consumption (total number of alcoholic drinks a participant consumed in a week; 1 drink being equivalent to 10 g of ethanol)^13^: 1 to 14 (women) or 1 to 21 (men) drinks per week (optimal), no alcohol (intermediate),^17^ and greater than or equal to 15 (women) or greater than or equal to 22 (men) drinks per week (poor).

We then computed an overall healthy lifestyle score by aggregating responses for the 4 individual lifestyle factors: optimal (2 points), intermediate (1 point), or poor (0 points). This scale resulted in a healthy lifestyle score ranging from 0 (lowest healthy score, highest risk) to 8 (highest healthy score, lowest risk).

Sixteen lifestyle profiles were based on the combinations of 4 dichotomized (optimal vs intermediate or poor) lifestyle factors. We assigned letters to the 16 profiles, with A referring to no optimal lifestyle factors; B to E, 1 optimal lifestyle factor; F to K, to different combinations of 2 optimal lifestyle factors; L to O, different combinations of 3 optimal lifestyle factors; and P, 4 optimal lifestyle factors. Participants with all 4 optimal lifestyle factors included those who were never smokers, had a BMI less than 25, were physically active, and consumed a moderate amount of alcohol. In addition, there were 4 different profiles with 3 optimal lifestyle factors, 6 profiles with 2 optimal lifestyle factors, 4 profiles with 1 optimal lifestyle factor, and 1 profile with no optimal factors. In a sensitivity analysis, we included never and moderate drinkers in the optimal alcohol consumption category.

Participants were linked to national registers for hospitalizations, prescription reimbursements, and vital status during the follow-up period. In some studies, data from 5 yearly clinical examinations or from annual surveys were also used. The outcomes of interest were incident type 2 diabetes (*International Statistical Classification of Diseases, 10th Revision* [*ICD-10*] code E11), nonfatal myocardial infarctions (*ICD-10* codes I21-I22) and coronary deaths (*ICD-10* codes I20-I25), stroke (*ICD-10* codes I60, I61, I63, and I64), cancers (*ICD-10* codes C00-C97), asthma (*ICD-10* codes J45-J46), and COPD exacerbations (J41, J42, J43, and J44). In subsidiary analyses, heart failure (*ICD-10* code I50) and dementia (*ICD-10* codes F00, F01, F02, F03, G30, and G31) were included.

Individuals with a record of any of these diseases at baseline were excluded from the analyses. We also excluded participants with a record of type 1 diabetes (*ICD-10* code E10) at baseline.

### Statistical Analysis

All analyses were conducted separately for men and women. In the main analysis, disease-free years were defined as the time between ages 40 and 75 years that an individual was free from a diagnosis of any of the 6 (8 in subsidiary analyses) chronic diseases examined. We chose age 40 years, as this is typically the age at which health checks, particularly cardiovascular disease, are initiated.^18,19,20^

To estimate the association between healthy lifestyle score and disease-free years, hazard ratios with 95% CIs for the first disease were calculated using flexible parametric survival models on the cumulative hazards scale.^21^ Using age as the timescale, restricted cubic splines with 0 to 4 internal knots (depending on the cohort) were fitted within these models to estimate the baseline hazard for each healthy lifestyle score. The 95% CIs for disease-free years were estimated via bootstrapping using 1000 independent replications. When there were fewer than 10 participants in a category of the score within a study, the corresponding result was removed from the calculations because this would cause statistical instability.

Disease-free years according to overall healthy lifestyle score, number of optimal healthy factors, and 16 lifestyle profiles were estimated conditional on survival to age 40 years without any of the 6 major noncommunicable diseases investigated.

We used a 2-stage analysis to combine the results for the healthy lifestyle score. Thus, effect estimates were first calculated for each study (the first stage), then the study-specific results were pooled using random effects meta-analysis (the second stage). Heterogeneity between cohort studies was assessed with the *I^2^* and τ statistics. We tested for dose-response associations using meta-regression. Owing to small numbers of participants in selected studies, the analyses of 16 lifestyle profiles and number of optimal healthy factors were conducted using a pooled data set, for which access to individual participant level data was available (Électricité de France-Gaz de France Employees; Health and Social Support; Helsinki Health Study; Whitehall II; Work, Lipids, and Fibrinogen Norrland; Finnish Public Sector; and Work, Lipids, and Fibrinogen Stockholm).

We repeated the main analyses with an alternative outcome that included heart failure and dementia in addition to the 6 diseases. To assess missing baseline values as a source of bias, we repeated the main analysis after imputing missing values in each cohort using the proc mi program in SAS, version 9.4 (SAS Institute Inc). To examine whether the association between healthy lifestyle and disease-free life-years was robust across socioeconomic status hierarchy, we stratified the analyses by categories of SES.

Two-sided *P* values were used with an α level of .05 for statistical significance. Data were analyzed using Stata/MP, version 15.1 (StataCorp) for Mac, packages stpm2, metan, and metareg.^22,23^ Imputation of missing data was performed using SAS, version 9.4 (SAS Institute Inc).

## Results

Individual-level data available comprised a total of 139 380 people (Figure). We excluded 13 545 individuals (9.7%) owing to missing data on age, sex, BMI, smoking, physical activity, alcohol consumption, or chronic diseases. In addition, 9792 individuals (7.0%) with a history of any of the 6 chronic diseases at baseline were omitted. Thus, the analytic sample comprised 116 043 participants with data on height, weight, smoking, physical activity, and alcohol consumption, and no history of cancer, coronary heart disease, stroke, diabetes, asthma, or COPD at baseline. Of the 116 043 people included in the analysis, the mean (SD) age was 43.7 (10.1) years and 70 911 were women (61.1%) (Table 1). People with a more advantageous healthy lifestyle score were younger and more likely to be of higher socioeconomic status (eTable in the Supplement).

**Table 1.** Baseline Characteristics of Participants of 12 Prospective Cohort Studies (IPD-Work Consortium)^a^

Study^a^	Baseline year	No.	Age, mean (SD), y	Distribution of healthy lifestyle score, %								
				0	1	2	3	4	5	6	7	8
**Men**												
COPSOQ II	2004-2005	2533	43.5 (11.1)	NA	1	3	7	14	21	22	20	11
DWECS 2000	2000	3856	42.1 (13.7)	NA	1	5	11	19	23	23	14	5
DWECS 2005	2005	2754	40.9 (12.9)	<1	1	4	10	16	22	22	16	8
FPS	2000	8085	44.8 (9.4)	<1	2	5	9	16	19	22	19	9
Gazel	1997	6381	51.0 (2.4)	<1	2	7	13	20	26	20	11	1
HeSSup	1998	7970	37.3 (11.4)	<1	1	4	8	15	17	23	18	13
HHS	2000-2001	1312	49.9 (6.6)	<1	1	4	8	14	19	23	19	12
IPAW	1996-1967	615	41.1 (10.0)	NA	NA	3	8	16	25	26	15	5
PUMA	1999-2000	301	44.3 (10.3)	NA	NA	NA	6	17	23	30	13	7
Whitehall II	1991-1993	4856	49.1 (6.0)	<1	<1	2	6	12	19	27	23	11
WOLF N	1996-1998	3527	43.7 (10.2)	<1	1	3	7	15	24	24	18	8
WOLF S	1992-1995	2942	41.5 (11.0)	<1	1	3	8	14	20	22	19	14
Total cohort	1991-2005	45 132	44.1 (9.8)	<1	1	4	9	16	21	23	17	9
**Women**												
COPSOQ II	2004-2005	2892	42.9 (11.0)	NA	1	2	6	13	20	24	21	12
DWECS 2000	2000	3986	41.9 (13.6)	NA	<1	3	9	20	26	22	16	4
DWECS 2005	2005	3088	40.7 (12.8)	NA	1	3	8	16	22	22	20	7
FPS	2000	34 209	44.3 (9.4)	<1	<1	2	5	11	18	24	24	15
Gazel	1997	2350	48.3 (3.7)	<1	<1	2	6	15	25	29	21	1
HeSSup	1998	11 966	36.0 (11.4)	<1	1	2	5	11	17	25	22	18
HHS	2000-2001	4862	49.1 (6.6)	<1	<1	2	5	11	17	24	23	18
IPAW	1996/7	1213	40.8 (10.5)	NA	NA	1	6	14	28	25	17	8
PUMA	1999-2000	1379	42.1 (10.0)	NA	NA	1	5	15	25	25	19	8
Whitehall II	1991-1993	2136	50.0 (6.1)	<1	1	3	10	16	24	22	17	7
WOLF N	1996-1998	661	44.1 (10.0)	0	<1	2	5	15	16	25	22	15
WOLF S	1992-1995	2169	40.9 (10.8)	<1	<1	2	5	11	19	22	24	16
Total cohort	1991-2005	70 911	43.7 (10.1)	<1	<1	2	6	12	19	24	22	14

Abbreviations: COPSOQ-II, Copenhagen Psychosocial Questionnaire study II; DWECS, Danish Work Environment Cohort Study; FPS, Finnish Public Sector Study; Gazel, Électricité de France-Gaz de France Employees; HeSSup, Health and Social Support; HHS, Helsinki Health Study; IPAW, Intervention Project on Absence and Well-being; IPD-Work, Individual-Participant-Data Meta-Analysis in Working Populations; NA, not available; PUMA, Burnout, Motivation and Job Satisfaction study; WOLF N, Work, Lipids and Fibrinogen Study, Norrland; WOLF S, Work, Lipids and Fibrinogen Study, Stockholm.

^a^ The IPD-Work studies included the Copenhagen Psychosocial Questionnaire study II, Denmark; the Danish Work Environment Cohort Studies from 2000 and 2005, Denmark; the Finnish Public Sector Study, Finland; a cohort study of Électricité de France-Gaz de France employees, France; the Health and Social Support Study, Finland; the Helsinki Health Study, Finland; the Intervention Project on Absence and Well-being study, Denmark; the Burnout, Motivation and Job Satisfaction study, Denmark; the Whitehall II Study, United Kingdom; the Work, Lipids and Fibrinogen Study, Norrland, Sweden; and the Work, Lipids and Fibrinogen Study, Stockholm, Sweden.

The mean follow-up duration in these analyses was 12.5 years (range between studies, 4.9-18.6 years) with 1.45 million person-years at risk. A total of 8012 of 45 132 men (17.8%) had at least 1 incident disease during 545 113 person-years at risk (incidence, 14.7 per 1000 person-years). The corresponding figure was 9371 of 70 911 women (13.2%) during 904 207 person-years at risk (incidence, 10.4 per 1000 person-years). A total of 17 383 participants developed at least 1 chronic disease.

According to separate meta-analyses for each healthy lifestyle score, men with zero points on the score had 21.7 (95% CI, 18.5-24.8) disease-free years between ages 40 and 75 years, while those with the maximum of 8 points had 30.9 (95% CI, 30.2-31.5) disease-free years (Table 2). The corresponding summary figures for women were 21.6 (95% CI, 17.7-25.6) and 30.7 (95% CI, 30.2-31.1). Comparing the best lifestyle score with the worst lifestyle score was associated with 9.9 (95% CI, 6.7-13.1) additional years without chronic diseases in men and 9.4 (95% CI, 5.4-13.3) additional years in women; owing to small numbers, this comparison was possible to calculate only for the 3 largest cohorts: Finnish Public Sector, Health and Social Support, and Électricité de France-Gaz de France Employees.

**Table 2.** Estimated Number of Disease-Free Life-Years and Age Achieved Disease Free by Level of Healthy Lifestyle Score^a^

Healthy lifestyle score	No. of cases (total)^b^	Disease free from age 40 y (95% CI), y	Age reached disease free, mean (95% CI), y
Men			
0	32 (84)	21.7 (18.5-24.8)	61.7 (58.5-64.8)
1	188 (570)	24.3 (23.0-25.5)	64.3 (63.0-65.5)
2	504 (1759)	25.2 (24.1-26.2)	65.2 (64.1-66.2)
3	930 (3760)	26.4 (25.6-27.3)	66.4 (65.6-67.3)
4	1429 (6592)	27.5 (26.9-28.0)	67.5 (66.9-68.0)
5	1615 (8629)	28.6 (28.0-29.2)	68.6 (68.0-69.2)
6	1612 (9534)	29.4 (28.9-30.0)	69.4 (68.9-70.0)
7	995 (7214)	30.2 (29.6-30.8)	70.2 (69.6-70.8)
8 (Healthiest)	349 (3521)	30.9 (30.2-31.5)	70.9 (70.2-71.5)
Women			
0	19 (54)	21.6 (17.7-25.6)	61.6 (57.7-65.6)
1	107 (326)	22.6 (20.1-25.1)	62.6 (60.1-65.1)
2	347 (1413)	25.4 (23.9-26.9)	65.4 (63.9-66.9)
3	868 (3961)	26.7 (25.8-27.6)	66.7 (65.8-67.6)
4	1502 (8614)	27.4 (26.6-28.1)	67.4 (66.6-68.1)
5	2022 (13 426)	28.5 (27.9-29.0)	68.5 (67.9-69.0)
6	2085 (17 205)	29.4 (28.8-29.9)	69.4 (68.8-69.9)
7	1565 (15 950)	30.4 (29.8-30.9)	70.4 (69.8-70.9)
8 (Healthiest)	841 (9863)	30.7 (30.2-31.1)	70.7 (70.2-71.1)

^a^ Disease-free life-years refer to the number of life-years between ages 40 and 75 years that an individual was free from a diagnosis of any of the following noncommunicable diseases: type 2 diabetes, coronary heart disease, stroke, cancer, asthma, and chronic obstructive pulmonary disease. Healthy lifestyle score included 4 lifestyle factors (smoking, body mass index, physical activity, and alcohol consumption) which were each allocated a score based on known risk status (0, 1, or 2) and then aggregated (range, 0-8).

^b^ Indicates the number of participants who developed 1 or more chronic diseases during follow-up.

The association between healthy lifestyle score and the number of disease-free life-years followed a dose-response association (*P* < .001 for both sexes); an increase of 1 point (advantage) was associated with an elevation of 0.96 (95% CI, 0.83-1.08) years in disease-free life-years in men and an increase of 0.89 (95% CI, 0.75-1.02) disease-free life-years in women (eFigure 1 in the Supplement). The association between healthy lifestyle score and years lived without chronic diseases remained unchanged after imputing missing baseline values and the association was observed in all socioeconomic groups (eFigures 2 and 3 in the Supplement). In the pooled data set of 93 426 participants, there was a linear association between the number of optimal lifestyle factors and disease-free years in the total sample and at all levels of socioeconomic status (eFigures 4, 5, and 6 in the Supplement).

Table 3 provides the number of disease-free years and age achieved without chronic disease according to 16 lifestyle profiles. The 4 lifestyle profiles that were associated with the highest number of disease-free years (profiles P, L, M, and N in men and women) included a BMI less than 25 and at least 2 health behaviors of never smoking, physical activity, and moderate alcohol consumption. Participants with these lifestyle profiles reached age 70.3 years (95% CI, 69.9-70.8) to 71.4 (95% CI, 70.9-72.0) years disease free (depending on the profile and sex). None of the 3 profiles associated with the shortest disease-free lifespan (profiles C, E, and A) included a BMI less than 25 or physical activity. Two of these adverse profiles included either never smokers (C) or moderate drinkers (E), but not both. Including nondrinkers and moderate drinkers in the optimal alcohol consumption category did not materially change the results (eFigure 7 in the Supplement).

**Table 3.** Estimated Number of Disease-Free Life-Years^a^ and Age Achieved Disease Free for 16 Lifestyle Profiles

No. of optimal lifestyle factors	Profile^b^	Optimal lifestyle factors				Disease free from age 40 y (95% CI), y		Age reached disease free, mean (95% CI), y	
		Normal weight	Never smoker	Physically active	Moderate alcohol use	Men	Women	Men	Women
0	A	No	No	No	No	27.2 (26.7-27.6)	27.9 (27.4-28.3)	67.2 (66.7-67.6)	67.9 (67.4-68.3)
1	B	Yes	No	No	No	28.9 (28.4-29.4)	30.1 (29.5-30.7)	68.9 (68.4-69.4)	70.1 (69.5-70.7)
	C	No	Yes	No	No	28.1 (27.0-29.2)	28.1 (27.9-28.4)	68.1 (67.0-69.2)	68.1 (67.9-68.4)
	D	No	No	Yes	No	28.5 (27.8-29.3)	29.1 (28.5-29.7)	68.5 (67.8-69.3)	69.1 (68.5-69.7)
	E	No	No	No	Yes	27.5 (27.2-27.9)	27.9 (27.5-28.4)	67.5 (67.2-67.9)	67.9 (67.5-68.4)
2	F	Yes	Yes	No	No	29.7 (29.1-30.3)	30.8 (30.3-31.2)	69.7 (69.1-70.3)	70.8 (70.3-71.2)
	G	Yes	No	Yes	No	29.7 (29.3-30.1)	30.5 (30.0-30.9)	69.7 (69.3-70.1)	70.5 (70.0-70.9)
	H	Yes	No	No	Yes	29.3 (28.9-29.6)	30.3 (30.1-30.4)	69.3 (68.9-69.6)	70.3 (70.1-70.4)
	I	No	Yes	Yes	No	29.7 (29.2-30.2)	29.2 (28.6-29.9)	69.7 (69.2-70.2)	69.2 (68.6-69.9)
	J	No	Yes	No	Yes	29.4 (29.1-29.7)	29.0 (28.8-29.3)	69.4 (69.1-69.7)	69.0 (68.8-69.3)
	K	No	No	Yes	Yes	28.5 (28.0-29.0)	28.9 (28.5-29.3)	68.5 (68.0-69.0)	68.9 (68.5-69.3)
3	L	Yes	Yes	Yes	No	30.9 (30.1-31.8)	31.4 (30.9-32.0)	70.9 (70.1-71.8)	71.4 (70.9-72.0)
	M	Yes	Yes	No	Yes	30.6 (30.2-30.9)	31.2 (30.9-31.4)	70.6 (70.2-70.9)	71.2 (70.9-71.4).
	N	Yes	No	Yes	Yes	30.3 (29.9-30.8)	31.1 (30.8-31.3)	70.3 (69.9-70.8)	71.1 (70.8-71.3)
	O	No	Yes	Yes	Yes	29.6 (29.2-30.0)	29.8 (29.4-30.2)	69.6 (69.2-70.0)	69.8 (69.4-70.2)
4	P	Yes	Yes	Yes	Yes	31.2 (30.9-31.6)	31.2 (30.9-31.5)	71.2 (70.9-71.6)	71.2 (70.9-71.5)

^a^ Disease-free life-years refer to the number of life-years between ages 40 and 75 years that an individual was free from a diagnosis of any of the following noncommunicable diseases: type 2 diabetes, coronary heart disease, stroke, cancer, asthma, and chronic obstructive pulmonary disease.

^b^ Sixteen lifestyle profiles include all combinations of having 0, 1, 2, 3, or 4 of the following optimal lifestyle factors: body mass index less than 25 (calculated as weight in kilograms divided by height in meters squared), never smoking, being physically active, and moderate alcohol consumption.

Defining the number of years without major chronic disease by the presence of heart failure and dementia in addition to the 6 diseases did not substantially change the results on healthy lifestyle score or 16 lifestyle profiles (Table 4 and eFigures 8, 9, and 10 in the Supplement).

**Table 4.** Age Achieved Free From 8 Chronic Diseases,^a^ Including Heart Failure and Dementia, by Lifestyle Profile

No. of optimal lifestyle factors	Profile^b^	Lifestyle profiles				Age achieved disease free, mean (95% CI), y
		Normal weight	Never smoker	Physically active	Moderate drinker	
**Men (n = 35 073)**						
4	P	Yes	Yes	Yes	Yes	70.6 (70.1-71.0)
3	L	Yes	Yes	Yes	No	70.2 (69.4-71.0)
3	M	Yes	Yes	No	Yes	69.9 (69.3-70.4)
3	N	Yes	No	Yes	Yes	69.6 (69.1-70.1)
2	F	Yes	Yes	No	No	68.8 (68.0-69.6)
2	G	Yes	No	Yes	No	68.8 (68.0-69.6)
3	O	No	Yes	Yes	Yes	68.7 (68.1-69.2)
2	J	No	Yes	No	Yes	68.6 (68.0-69.1)
2	I	No	Yes	Yes	No	68.5 (67.5-69.5)
2	H	Yes	No	No	Yes	68.3 (67.7-68.8)
1	B	Yes	No	No	No	67.5 (66.8-68.3)
2	K	No	No	Yes	Yes	67.5 (67.0-68.0)
1	D	No	No	Yes	No	67.4 (66.7-68.2)
1	C	No	Yes	No	No	67.1 (66.2-68.0)
1	E	No	No	No	Yes	66.5 (66.0-67.0)
0	A	No	No	No	No	66.0 (65.4-66.7)
**Women (n = 58 353)**						
4	P	Yes	Yes	Yes	Yes	71.0 (70.7-71.3)
3	L	Yes	Yes	Yes	No	70.9 (70.3-71.4)
3	M	Yes	Yes	No	Yes	70.8 (70.5-71.2)
3	N	Yes	No	Yes	Yes	70.8 (70.5-71.1)
2	F	Yes	Yes	No	No	70.3 (69.8-70.9)
1	B	Yes	No	No	No	69.8 (69.2-70.5)
2	G	Yes	No	Yes	No	69.8 (69.2-70.4)
2	H	Yes	No	No	Yes	69.8 (69.5-70.2)
3	O	No	Yes	Yes	Yes	69.3 (68.8-69.8)
2	I	No	Yes	Yes	No	68.9 (68.1-69.7)
2	J	No	Yes	No	Yes	68.7 (68.2-69.1)
2	K	No	No	Yes	Yes	68.5 (68.1-69.0)
1	D	No	No	Yes	No	68.4 (67.6-69.2)
1	C	No	Yes	No	No	67.7 (67.1-68.4)
1	E	No	No	No	Yes	67.3 (66.8-67.7)
0	A	No	No	No	No	67.1 (66.5-67.8)

^a^ The 8 chronic diseases were the following noncommunicable diseases: type 2 diabetes, coronary heart disease, stroke, cancer, asthma, and chronic obstructive pulmonary disease, as well as heart failure and dementia.

^b^ Labeling of profiles as in Table 3.

## Discussion

The main finding of this study was that a high overall healthy lifestyle score and various lifestyle profiles characterized by 4 optimal lifestyle factors were associated with significant gains in years lived without major noncommunicable diseases between ages 40 and 75 years in both sexes. Comparing the best with the worst lifestyle score was associated with approximately 9 additional years without chronic diseases. A 1-point advantage in healthy lifestyle score was associated with an almost 1-year increase in years spent without type 2 diabetes, coronary heart disease, stroke, cancer, asthma, and COPD. Of the 16 different lifestyle profiles studied, all 4 that were associated with the longest disease-free life span included a BMI less than 25 and at least 2 of the following health behaviors: never smoking, physical activity, and moderate alcohol consumption. The results were essentially the same when heart failure and dementia—2 further common conditions of older age—were considered in addition to the other 6 diseases.

We are not aware of other large-scale investigations on the different combinations of common lifestyle factors and disease-free life-years. Our findings suggest that normal weight is a particularly important component of the lifestyle profiles, although a greater total number of optimal lifestyle factors also characterized individuals who achieved a higher age without chronic disease. Our findings do not support a synergistic role for any specific combination of lifestyle factors; rather, a normal BMI, never smoking, physical activity, and moderate alcohol consumption appear to be associated with health span in a way that is consistent with an additive effect.

Our results regarding overall lifestyle score are comparable to those reported in previous studies on disease-free years and healthy lifestyle factors using heterogeneous operationalizations of the exposure and outcome.^3,4,5^ For example, a study of 33 000 men and women aged 20 to 70 years^3^ found approximately 2 extra disease-free years from chronic diseases in participants with all vs none of nonsmoking status, BMI less than 25, physical activity, and adherence to a Mediterranean-style diet (excluding alcohol). In a pooled analysis of 4 cohort studies,^4^ participants who were not smokers, physically inactive, or obese lived several years longer without 4 chronic diseases than those with at least 2 of these risk factors. In a general population sample,^5^ the combination of absence of smoking, hypertension, and overweight was associated with a substantial delay in the onset of stroke, heart disease, diabetes, chronic respiratory disease, cancer, and neurodegenerative disease.

Our findings are biologically plausible. Obesity is associated with elevated blood pressure, insulin resistance, and dyslipidemia, increasing the risk of cardiometabolic diseases. In addition, increased fat mass in the chest and abdomen causes reduction of lung volume and alteration in the pattern of ventilation, affecting respiratory function and increasing the odds of site-specific cancer.^24,25^ Higher BMI has been associated with lower rather than higher risk for COPD, but long-term follow-up suggests that this increased risk may be attributable to the effects of undiagnosed or preclinical COPD, which lead to weight loss.^26^ Evidence of a causal relationship between adult BMI and the risk of asthma has been supported by a recent Mendelian randomization study.^27^

The health benefits of regular physical activity include reductions in blood pressure, lower systemic inflammation and abdominal adiposity, and improvements in insulin sensitivity and lipid lipoprotein profiles.^28^ Physical activity may prevent type 2 diabetes, heart and pulmonary diseases, and cancer.^29^ The mechanisms associating compounds inhaled from tobacco smoke with cancer, cardiovascular diseases, and pulmonary diseases include DNA damage, inflammation, and oxidative stress.^30^ Alcohol affects health via intoxication, glucose metabolism, inflammation, and other mechanisms; moderate alcohol use has been associated with a lower risk for some disease outcomes, including coronary heart disease, diabetes, and COPD, while the risk of cancer appears to be lower in association with the less the person consumes alcohol.^31,32^

### Limitations

Although our study has its strengths, including its scale and focus on specific combinations of healthy lifestyle factors, it also has several limitations. In the present study, only 3002 participants (2.6% of the total study population) died during the follow-up, precluding analyses of life expectancy. In addition, we limited estimation of disease-free years to between ages 40 and 75 years. Although this limit accords with other studies^4,15^ and corresponds to recommended age ranges for risk calculators used in clinical practice (eg, the Framingham Cardiovascular Risk Score, the American College of Cardiology/American Heart Association Guideline on the Assessment of Cardiovascular Risk and the European SCORE),^20,33,34^ further research covering the entire health and life span would be informative.

The possibility of confounding cannot be excluded in observational studies, although confounding by socioeconomic influences is an unlikely explanation for current findings, as the results were replicable across socioeconomic hierarchy. Our exposure was based on self-reported measurement when, at least for smoking and physical activity, useful biomarkers and wearable biomonitoring techniques are available. We also relied on a single baseline assessment of our exposures, which therefore does not allow for the exploration of the association with adoption of healthy behaviors. Despite careful harmonization of the variables, the crude measurements and variation in questionnaires between participating cohort studies could have led to some misclassification and heterogeneity in study-specific estimates. We were unable to assess dietary habits, which is an important lifestyle factor, although BMI was used as a proxy of excessive calorific intake.

In addition to the exclusion of participants with prevalent disease at baseline, which may have diluted the difference by unevenly affecting those with unhealthy habits who had already developed some disease, most of the studies in our pooled analysis were occupational cohorts, which include healthier people than the general population. However, empirical analyses suggest no difference in risk factor-mortality associations between these 2 samples.^35^ In addition, we did not have genetic material with which to determine the role of genetic factors in the association between lifestyle and disease-free life-years.

## Conclusions

The results of this study suggest a consistent dose-response association of a higher number of healthy lifestyle factors with the number of disease-free years both in men and women and across the socioeconomic strata, and that various healthy lifestyle profiles, particularly those including a BMI less than 25, are associated with a prolonged health span. These findings may be useful for prevention, strengthening the evidence base for actions to support healthy choices in everyday life.

## Notes

Supplement. **eMethods.** Detailed Methods **eTable.** Baseline Characteristics of Participants According to Levels of Healthy Lifestyle Score (IPD-Work Consortium) **eFigure 1.** Meta-regression of Linear Trend Between Healthy Lifestyle Score and Disease-Free Years Between Ages 40 and 75 **eFigure 2.** Number of Disease-Free Life-Years and Age Achieved Disease-Free by Level of Healthy Lifestyle Score After Imputing Missing Predictor Values **eFigure 3.** Number of Disease-Free Life-Years and Age Achieved Disease-Free by Level of Healthy Lifestyle Score and Socioeconomic Status **eFigure 4.** Number of Disease-Free Life-Years and Age Achieved Disease-Free by Number of Optimal Lifestyle Factors **eFigure 5.** Number of Disease-Free Life-Years and Age Achieved Disease-Free by Number of Optimal Lifestyle Factors by Socioeconomic Status **eFigure 6.** Meta-regression of Linear Trend Between Number of Optimal Lifestyle Factors Score and Disease-Free Years Between Ages 40 and 75 **eFigure 7.** Number of Disease-Free Life-Years and Age Achieved Disease-Free for 16 Lifestyle Profiles With Revised Optimal Alcohol Consumption Including Non-Drinkers and Moderate Drinkers **eFigure 8.** Number of Disease-Free Life-Years and Age Achieved Disease-Free by Level of Healthy Lifestyle Score After Including Two Additional Disease Endpoints (Heart Failure and Dementia) **eFigure 9.** Number of Disease-Free Life-Years and Age Achieved Disease-Free by Number of Optimal Lifestyle Factors After Including Two Additional Disease Endpoints (Heart Failure and Dementia) **eFigure 10.** Number of Disease-Free Life-Years and Age Achieved Disease-Free for 16 Lifestyle Profiles After Including Two Additional Disease Endpoints (Heart Failure and Dementia) **eReferences**
