# Supplementary material for: Association of Healthy Lifestyle With Years Lived Without Major Chronic Diseases
Source: JAMA Intern Med. 2020 Apr 6;180(5):1–10. doi: 10.1001/jamainternmed.2020.0618 (PMC7136858; doi:10.1001/jamainternmed.2020.0618)
Supplement: Supplement. — eMethods. Detailed Methods eTable. Baseline Characteristics of Participants According to Levels of Healthy Lifestyle Score (IPD-Work Consortium) eFigure 1. Meta-regression of Linear Trend Between Healthy Lifestyle Score and Disease-Free Years Between Ages 40 and 75 eFigure 2. Number of Disease-Free Life-Years and Age Achieved Disease-Free by Level of Healthy Lifestyle Score After Imputing Missing Predictor Values eFigure 3. Number of Disease-Free Life-Years and Age Achieved Disease-Free by Level of Healthy Lifestyle Score and Socioeconomic Status eFigure 4. Number of Disease-Free Life-Years and Age Achieved Disease-Free by Number of Optimal Lifestyle Factors eFigure 5. Number of Disease-Free Life-Years and Age Achieved Disease-Free by Number of Optimal Lifestyle Factors by Socioeconomic Status eFigure 6. Meta-regression of Linear Trend Between Number of Optimal Lifestyle Factors Score and Disease-Free Years Between Ages 40 and 75 eFigure 7. Number of Disease-Free Life-Years and Age Achieved Disease-Free for 16 Lifestyle Profiles With Revised Optimal Alcohol Consumption Including Non-Drinkers and Moderate Drinkers eFigure 8. Number of Disease-Free Life-Years and Age Achieved Disease-Free by Level of Healthy Lifestyle Score After Including Two Additional Disease Endpoints (Heart Failure and Dementia) eFigure 9. Number of Disease-Free Life-Years and Age Achieved Disease-Free by Number of Optimal Lifestyle Factors After Including Two Additional Disease Endpoints (Heart Failure and Dementia) eFigure 10. Number of Disease-Free Life-Years and Age Achieved Disease-Free for 16 Lifestyle Profiles After Including Two Additional Disease Endpoints (Heart Failure and Dementia) eReferences [file jamainternmed-180-760-s001.pdf]

Supplementary Online Content

Nyberg ST, Singh-Manoux A, Pentti J, et al. Association of healthy lifestyle with years lived without major chronic diseases. *JAMA Intern Med*. Published online April 6, 2020. doi:10.1001/jamainternmed.2020.0618

eMethods. Detailed Methods

eTable. Baseline Characteristics of Participants According to Levels of Healthy Lifestyle Score (IPD-Work Consortium)

eFigure 1. Meta-regression of Linear Trend Between Healthy Lifestyle Score and Disease-Free Years Between Ages 40 and 75

eFigure 2. Number of Disease-Free Life-Years and Age Achieved Disease-Free by Level of Healthy Lifestyle Score After Imputing Missing Predictor Values

eFigure 3. Number of Disease-Free Life-Years and Age Achieved Disease-Free by Level of Healthy Lifestyle Score and Socioeconomic Status

eFigure 4. Number of Disease-Free Life-Years and Age Achieved Disease-Free by Number of Optimal Lifestyle Factors

eFigure 5. Number of Disease-Free Life-Years and Age Achieved Disease-Free by Number of Optimal Lifestyle Factors by Socioeconomic Status

eFigure 6. Meta-regression of Linear Trend Between Number of Optimal Lifestyle Factors Score and Disease-Free Years Between Ages 40 and 75

eFigure 7. Number of Disease-Free Life-Years and Age Achieved Disease-Free for 16 Lifestyle Profiles With Revised Optimal Alcohol Consumption Including Non-Drinkers and Moderate Drinkers

eFigure 8. Number of Disease-Free Life-Years and Age Achieved Disease-Free by Level of Healthy Lifestyle Score After Including Two Additional Disease Endpoints (Heart Failure and Dementia)

eFigure 9. Number of Disease-Free Life-Years and Age Achieved Disease-Free by Number of Optimal Lifestyle Factors After Including Two Additional Disease Endpoints (Heart Failure and Dementia)

eFigure 10. Number of Disease-Free Life-Years and Age Achieved Disease-Free for 16 Lifestyle Profiles After Including Two Additional Disease Endpoints (Heart Failure and Dementia)

eReferences

This supplementary material has been provided by the authors to give readers additional information about their work.

© 2020 Nyberg ST et al. *JAMA Internal Medicine.*

eMethods. Detailed Methods

Description of IPD-Work Cohort Studies

The studies included in this study were the Copenhagen Psychosocial Questionnaire study II, Denmark (COPSOQ-II), the Danish Work Environment Cohort Studies from 2000 and 2005, Denmark (DWECS), the Finnish Public Sector Study, Finland (FPS), a cohort study of Électricité de France-Gaz de France employees, France (Gazel), the Health and Social Support Study, Finland (HeSSup), the Helsinki Health Study, Finland (HHS), the Intervention Project on Absence and Well-being study, Denmark (IPAW), the Burnout, Motivation and Job Satisfaction study, Denmark (PUMA), the Whitehall II Study, the UK (Whitehall II), the Work, Lipids and Fibrinogen Study, Norrland, Sweden (WOLF N), and the Work, Lipids and Fibrinogen Study, Stockholm, Sweden (WOLF S).

Copenhagen Psychosocial Questionnaire version II (COPSOQ II)[1]

COPSOQ II was carried out in 2004-2005. It included a follow up of respondents from COPSOQ I and also a representative sample of Danish residents aged 20-60 at study baseline. The questionnaire was sent to 8 000 individuals from the random sample and 4 732 individuals responded. With respondents, who were originally invited to COPSOQ-I, the total number of participants with a linkage to register data was 5998. The questionnaire could be completed using the posted questionnaire or via internet. COPSOQ-II was approved by and registered with the Danish Data protection agency (registration number: 2004-54-1493). Data on occupational position were obtained through linkage to a national register (the Employment Classification Module).

Self-reported height, weight and smoking status were collected from the questionnaires. Physical activity was based on responses from the questionnaire to the question: “This question is about physical exercise during leisure time. Which description fits best on you?”. Those stating that they were “Mostly physically passive or light physical activity for less than 2 hours per week (eg. reading, television, movies)” were classified as physically inactive, while those responding that they engaged in “Light physical activity for 2-4 hours per week (eg. walking, light garden work, light exercise)” were considered intermediate and those responding that they engaged in “Light physical activity for more than 4 hours per week or more strenuous activity for 2-4 hours per weeks (eg. walking/cycling in a fast speed, heavy garden work, exercising where you are getting either breathless or sweating)” or “More strenuous physical activity for more than 4 hours or regular hard exercise/competition several times per week” were regarded as physically active.

Weekly alcohol consumption was measured using a single item concerning average weekly alcohol consumption with separate responses for number of bottles of beer, glasses of wine or units of liqueur per week. For the present analysis the types of alcohol were summed to obtain weekly alcohol consumption.

Danish Work Environment Cohort Study (DWECS), Denmark[2,3]

DWECS is a split panel survey of working age Danish people. The cohort was established in 1990, when a simple random sample of men and women, aged 18-59, was drawn from the Danish population register. The participants have been followed up at five-year intervals and data from the years 2000 and 2005 were used for the present analysis. In 2000, 11 437 individuals were invited to participate and 8583 agreed to do so. 8552 of them were successfully linked with register data. In 2005, an additional random sample of 8545 individuals were invited to participate of which 5176 agreed to do so. First time respondents originally invited for DWECS 2000 but who did not respond that time, were additionally included in this sample with a total of 6741 participants successfully linked with registers. In Denmark, questionnaire- and register-based studies do not require ethics committee approval. DWECS was approved by and registered with the Danish Data protection agency (registration number: 2007-54-0059).

Self-reported height, weight and smoking status were collected from the questionnaires. For DWECS 2000, physical activity was based on responses from the questionnaire to the question “Which group are you to be placed in if you should describe your physical activity (including transport to and from work) in your spare time within the last 12 months?”. Those responding “Almost physically passive or light physical activity for less than two hours per week (e.g. reading, watching TV, cinema)” were classified as physically inactive, while those responding that they engaged in “Light physical activity, two-four hours per week (walk, cycle, light garden work, light exercises)” were considered intermediate while those responding “Light physical activity for more than four hours per week or more exhausting physical activity for two-four hours per week (e.g. fast walking and/or fast cycling (catch up with other cyclists), heavy garden work, exhausting exercises (you sweat and become short of breath)” or “Physically exhausting activity for more than four hours per week or regular, exhausting exercises and maybe competition several times per week” were considered physically active. Daily alcohol consumption was measured using the item: How much alcohol do you averagely drink per day?” asking the respondent to indicate the number of bottles of beer, glasses of wine, and units of other alcoholic liquors per day. These responses were added and multiplied by 7 to yield weekly alcohol consumption.

For DWECS 2005, physical activity was measured using the item “Looking at the past year, how would you best describe your level of leisure time activity?”. We regarded the response “Reading, watching tv, going to the cinema or other sedentary occupation or light physical activity for less than 2 hours per week” ” as physically inactive, while

© 2020 Nyberg ST et al. *JAMA Internal Medicine.*

those responding that they engaged in “Walking, cycling or other light exercise for 2-4 hours per week (also count Sunday trips, light gardening and cycling/walking to work)” were considere intermediate, and the responses “Doing heavy exercise, brisk walking/cycling or heavy gardening at least 4 hours per week or more strenuous physical activity 2-4 hours per week”, and “Heavy training and competition sports regularly and several times a week (more than 4 hours per week)” were regarded physically active. Alcohol was measured using two items regarding average daily alcohol consumption during weekdays and average daily alcohol consumption during the weekend, respectively. The number of alcoholic units were multiplied by 5 for weekdays and 2 for weekends and then summed to obtain weekly alcohol consumption.

Finnish Public Sector study (FPS), Finland[4]
The Finnish Public Sector study is a prospective cohort study comprising the entire public sector personnel of 10 towns (municipalities) and 21 hospitals in the same geographical areas. Participants, who were recruited from employers' records in 2000-2002, were individuals who had been employed in the study organisations for at least six months prior to data collection. 48 592 individuals (9 337 men and 39 255 women aged 17 to 65) responded to the questionnaire. Ethical approval was obtained from the ethics committee of the Finnish Institute of Occupational Health.

Self-reported height, weight and smoking status were collected from the questionnaires. Physical activity and alcohol consumption were based on responses from the questionnaire. The weekly amount of walking, brisk walking, jogging or running was asked with the following categories “not at all”, “less than half an hour”, “approximately one hour”, “2-3 hours” and “4 hours of more”. Physical inactivity was defined as less than 0.5 hour of each (brisk walking, jogging or running) per week. The weekly amount of both moderate and vigorous activities was calculated for classifying moderate or optimal activity. Alcohol consumption was based on the reported amounts of beer, wine or other mild alcoholic beverages and hard liquors. For each category, seven pre-defined answer alternatives were given and weekly consumption was estimated based on the responses.

Gazel, France[5]
Gazel is a prospective cohort study of 20 625 employees (15 011 men and 5 614 women) of France's national gas and electricity company, Electricité de France-Gaz de France (EDF-GDF). Since the study baseline in 1989, when the participants were aged 35–50 years, they have been posted an annual follow-up questionnaire to collect data on health, lifestyle, individual, familial, social, and occupational factors. Gazel in 1997 was treated as a baseline year for our analyses. 11 448 individuals participated that year. The GAZEL study received approval from the national commission overseeing ethical data collection in France (Commission Nationale Informatique et Liberté).

Self-reported height, weight and smoking status were collected from the questionnaires. Physical activity and alcohol consumption were based on responses from the questionnaire. Physical activity was enquired with the following response alternatives: Yes, competitively (optimal), regularly at least once a week (intermediate), occasionally (intermediate) or No (inactive). Regarding alcohol consumption, the participant was asked whether or not he consumed wine, beer/cider or aperitifs/digestives during the previous week. For each, the number of days and maximum quantity per day with given response alternatives was asked. Weekly consumption of alcohol was based on the responses.

Health and Social Support (HeSSup), Finland[6]
The Health and Social Support (HeSSup) study is a prospective cohort study of a stratified random sample of the Finnish population in the following four age groups: 20–24, 30–34, 40–44, and 50–54. The participants were identified from the Finnish population register and posted an invitation to participate, along with a baseline questionnaire, in 1998. 25 898 individuals responded to the questionnaire in 1998. The Turku University Central Hospital Ethics Committee approved the study.

Self-reported height, weight and smoking status were collected from the questionnaires. Physical activity and alcohol consumption were based on responses from the questionnaire. The weekly amount of walking, brisk walking, jogging or running was asked with the following categories “not at all”, “less than half an hour”, “approximately one hour”, “2-3 hours” and “4 hours of more”. Physical inactivity was defined as less than 0.5 hour of each (brisk walking, jogging or running) per week. The weekly amount of both moderate and vigorous activities was calculated for classifying moderate or optimal activity. Alcohol consumption was based on the reported amounts of beer, wine or other mild alcoholic beverages and hard liquors. For each category, seven pre-defined answer alternatives were given and weekly consumption was estimated based on the responses.

Helsinki Health Study (HHS), Finland[7]
The Finnish Helsinki Health Study (HHS) is a prospective cohort study comprising all employees of the City of Helsinki, who turned 40, 45, 50, 55, or 60 years in 2000-2002. We included in this study all participants who responded to the baseline survey (n=8960, response rate 67%, 80% women) and provided an informed written consent to combine their survey responses with retrospective and prospective registerbased follow-up data on different diseases and mortality (n=6603). Ethical approvals for this study were obtained from the ethics committees of the health authorities of the City of Helsinki, and the Department of Public Health, University of Helsinki.

© 2020 Nyberg ST et al. *JAMA Internal Medicine.*

Self-reported height, weight and smoking status were collected from the questionnaires. Leisure-time physical activity (including commuting) and alcohol consumption were based on responses from the questionnaire. The weekly amount of walking, brisk walking, jogging or running was asked with the following categories "not at all", "less than half an hour", "0.5-1 hour", "2-3 hours" and "4 hours of more". Physical inactivity was defined as less than 0.5 hour of each (brisk walking, jogging or running) per week. The weekly amount of both moderate and vigorous activities was calculated for classifying moderate or optimal activity. Alcohol consumption was based on the reported amounts of beer/cider, wine or other mild alcoholic beverages and hard liquors. For each category, seven pre-defined answer alternatives were given and weekly consumption was estimated based on the responses.

Intervention Project on Absence and Well-being (IPAW), Denmark[8]

IPAW is a 5-year psychosocial work environment intervention study including 22 intervention and 30 control work places in three organisations (a large pharmaceutical company, municipal technical services and municipal nursing homes) in Copenhagen, Denmark. The baseline questionnaire was posted to all the employees at the selected work-sites between 1996 and 1997. Of the 2 721 employees who worked at the 52 IPAW sites, 2 068 men and women completed the baseline questionnaire and 2055 were successfully linked with register data. Interventions took place at 22 workplaces during 1996-98 at the organisational and interpersonal level. IPAW was approved by and registered with the Danish Data Protection Agency (registration number: 2000-54-0066).

Self-reported height, weight and smoking status were collected from the questionnaires. Physical activity was based on responses from the questionnaire using the item "Which group are you to be placed in if you should describe your physical activity (including transport to and from work) in your spare time within the last 12 months?". Those responding "Almost physically passive or light physical activity for less than two hours per week (e.g. reading, watching TV, cinema)" were classified as physically inactive, while those responding that they engaged in "Light physical activity, two-four hours per week (walk, cycle, light garden work, light exercises)" were classified as intermediate, while those engaging in "Light physical activity for more than four hours per week or more exhausting physical activity for two-four hours per week (e.g. fast walking and/or fast cycling (catch up with other cyclists), heavy garden work, exhausting exercises (you sweat and become short of breath)" or "Physically exhausting activity for more than four hours per week or regular, exhausting exercises and maybe competition several times per week" were regarded physically active.

Weekly alcohol consumption was measured using the item "How much alcohol have you drunk on average on a weekly basis during the past year?" asking the respondent to indicate the number of bottles of beer, glasses of wine, and units of other alcoholic liquors per week.

Burnout, Motivation and Job Satisfaction study (PUMA)[9]

Burnout, Motivation and Job Satisfaction study (Danish acronym: PUMA) is an intervention study of burn-out among employees in the human service sector. Selection criteria for the participating organizations was that they had between 200 and 500 employees, that occupational groups within each organization were willing to participate and that the organizations would commit to the entire five-year study period. Participants gave consent to having their national identity numbers collected and used in later record linkages to Danish hospitalization and cause of death registries (Hospitalsindlæggelsesregisteret, Dødsårsagsregisteret). At study baseline in 1999-2000, 1 914 participants agreed to take part, of whom 1 905 were successfully linked with the registers. PUMA was approved by the Scientific Ethical Committees (Videnskabsetisk Komiteer) in the counties in which the study was conducted and approved by and registered with the Danish Data Protection Agency (registration number: 2000-54-0048).

Self-reported height, weight and smoking status were collected from the questionnaires. Physical activity was based on responses from the questionnaire using the item "Which group are you to be placed in if you should describe your physical activity (including transport to and from work) in your spare time within the last 12 months?". Those responding "Almost physically passive or light physical activity for less than two hours per week (e.g. reading, watching TV, cinema)" were classified as physically inactive, while those responding that they engaged in "Light physical activity, two-four hours per week (walk, cycle, light garden work, light exercises)" were considered intermediate, and those engaging in "Light physical activity for more than four hours per week or more exhausting physical activity for two-four hours per week (e.g. fast walking and/or fast cycling (catch up with other cyclists), heavy garden work, exhausting exercises (you sweat and become short of breath)" or "Physically exhausting activity for more than four hours per week or regular, exhausting exercises and maybe competition several times per week" were combined as physically active.

Weekly alcohol consumption was measured using the item "How much alcohol have you drunk on average on a weekly basis during the 4 weeks?" asking the respondent to indicate the number of bottles of beer, glasses of wine, and units of other alcoholic liquors per week.

Whitehall II, UK[10]

The Whitehall II study is a prospective cohort study set up to investigate socioeconomic determinants of health. At study baseline in 1985-1988, 10 308 civil service employees (6 895 men and 3 413 women) aged 35-55 and working in 20 civil service departments in London were invited to participate in the study. The Whitehall II study protocol was approved by

© 2020 Nyberg ST et al. *JAMA Internal Medicine.*

the University College London Medical School committee on the ethics of human research. Written informed consent was obtained at each data collection wave.

Participants underwent a clinical examination where their height and weight were measured at the baseline by a clinical staff member. Self-reported smoking status was collected from the questionnaire. Physical activity and alcohol consumption were based on responses from the questionnaire. Weekly hours spent in moderately energetic or vigorous sport activities were requested. Physical inactivity was defined as “no moderate or vigorous exercise”. Otherwise the weekly amount of both moderate and vigorous activities was used for classifying moderate or optimal activity.

Units of alcohol consumed (spirits, wines, beer) during the last seven days was enquired and weekly consumption was calculated as a sum of the reported amounts.

WOLF (Work, Lipids, and Fibrinogen) Stockholm and WOLF Norrland studies, Sweden[11,12]

The WOLF (Work, Lipids, and Fibrinogen) Stockholm study is a prospective cohort study of 5 698 people (3 239 men and 2 459 women) aged 19–70 and working in companies in Stockholm county. WOLF Norrland is a prospective cohort of 4 718 participants aged 19-65 working in companies in Jämtland and Västernorrland counties. At study baseline the participants underwent a clinical examination and completed a set of health questionnaires. For WOLF Stockholm, the baseline assessment was undertaken at 20 occupational health units between November 1992 and June 1995 and for WOLF Norrland at 13 occupational health service units in 1996-98. The Regional Research Ethics Board in Stockholm, and the ethics committee at Karolinska Institutet, Stockholm, Sweden approved the study.

Participants underwent a clinical examination where their height and weight were measured at the baseline by a clinical staff member. Self-reported smoking status was collected from the questionnaire. Physical activity and alcohol consumption were based on responses from the questionnaire. “No or very little exercise, only occasional walks” was classified as physical inactivity, occasional exercise was classified as moderate and regular exercise as optimal physical activity. The frequency and amount of drinking beer / strong beer / wine / strong wine / spirits was requested and weekly alcohol consumption was derived from the responses.

© 2020 Nyberg ST et al. *JAMA Internal Medicine.*

Assessment of Baseline Lifestyle Factors

We calculated BMI using height and weight (weight in kilograms divided by height in meters squared), which were measured (in Whitehall II, WOLF N, and WOLF S) or self-reported (in COPSOQ II, DWECS 2000 and 2005, IPAW, FPS, Gazel, HeSSup, HHS, and PUMA). Participants with BMI values <15 or >50 kg/m^2 as probable outliers or erroneous values were excluded from the analyses. Information on smoking, leisure-time physical activity, and alcohol consumption was extracted from questionnaires completed by participants in all studies.[13-16]

Assessment of Age, Sex and Socioeconomic Status

Information on sex and age was obtained from population registries or interview (COPSOQ II, DWECS 2000 and 2005, HHS, IPAW, FPS, Gazel, PUMA, WOLF N, WOLF S) or from questionnaires completed by participants (HeSSup, Whitehall II). SES was based on occupational title obtained from employers' or other registers or questionnaires completed by participants and categorised into low, intermediate, or high. In HeSSup, SES was based on the participant's self-reported highest educational qualification. Participants who were self-employed or who had missing data on job title were included in the analyses in the "other" category for SES.

Ascertainment of Major Non-communicable Diseases

Participants were linked to national registers of hospitalisations, prescription reimbursements and mortality. In Whitehall II, participants additionally attended to 5-yearly clinical examinations. Data from annual surveys during the follow-up were available for the participants of the Gazel study.

Linked records of major chronic diseases covered both baseline and follow-up. The outcome of interest in the present study was the first record of either incident type 2 diabetes, coronary heart disease, stroke, cancer, asthma or COPD. These specific diseases were selected because they are the commonest major non-communicable diseases in developed countries[17,18] and targets prioritised for global disease prevention by the WHO.

Incident type 2 diabetes was defined as the first record of diagnosis corresponding to ICD-10 code E11. We collected records from hospital admissions and discharge registers and mortality registers with a mention of diagnosis of type 2 diabetes in any of the diagnosis codes. Additionally, in the Finnish datasets (FPS, HeSSup and HHS), participants were also defined as an incident type 2 diabetes case the first time they appeared in the nationwide drug reimbursement register as eligible for type 2 diabetes medication.[19] In the Whitehall II study, type 2 diabetes was ascertained by 2-h oral glucose tolerance test administered every 5 years[20] using World Health Organization criteria and complemented by self-reports of diabetes diagnosis and medication.[21] In the Gazel study, non-fatal cases were based on self-report from annual questionnaires.

Coronary heart disease events were identified from hospital discharge and mortality registers, annual self-report questionnaires, or clinical screening using WHO Multinational Monitoring of Trends and Determinants in Cardiovascular Disease (MONICA) Project criteria. We included all non-fatal myocardial infarctions that were recorded as I21–I22 (ICD-10) or 410 (ICD-9) and coronary deaths recorded as I20–I25 (ICD-10) and 410–414 (ICD-9) in any of the diagnose codes.

Incident stroke was defined with hospital and mortality records (I60, I61, I63, I64 in ICD-10; 430, 431, 433, 434, 436 in ICD-9).[22,23] In the Gazel study, non-fatal stroke cases were based on self-report from annual questionnaires.

Cancers, C00–C97 (ICD-10 any cancer), were identified via national cancer or mortality registers, except for Gazel, in which incident cancer events were ascertained from the employer's medical register and by confirming any self-reported cancer diagnosis with the participant's physician.[24]

Severe asthma (J45 or J46 in ICD-10 or 493 in ICD-9) and COPD exacerbations (J41, J42, J43, and J44 in ICD-10, or 491, 492, and 496 in ICD-9) were ascertained from hospital discharge and death registers in all studies except for Gazel, in which non-fatal asthma events were based on self-report from annual questionnaires and non-fatal COPD was not available.[25,26]

Participants with missing data on these outcomes and those with a record of any of these diseases already at baseline were excluded from the analyses. We also excluded participants with a record of type 1 diabetes at baseline: E10 (ICD-10) or 250 (ICD-9 and ICD-8).[23]

© 2020 Nyberg ST et al. *JAMA Internal Medicine.*

eTable. Baseline Characteristics of Participants According to Levels of Healthy Lifestyle Score (IPD-Work Consortium)

	Healthy Lifestyle Score								
	0	1	2	3	4	5	6	7	8
**Men**									
Mean age (SD)	45.9 (9.2)	47.5 (8.4)	46.4 (9.6)	46.1 (9.5)	45.5 (10.2)	45.2 (10.4)	44.1 (10.8)	42.2 (10.9)	38.9 (11.5)
Mean BMI (SD)	32.7 (2.8)	29.9 (3.6)	28.2 (4.4)	27.7 (3.9)	26.6 (3.7)	26 (3.2)	25.1 (2.8)	24.1 (2.3)	22.9 (1.5)
BMI category									
Normal weight	0 (0)	0 (0)	381 (20)	764 (19)	2163 (30)	3319 (35)	5241 (51)	5413 (69)	3857 (100)
Overweight	0 (0)	317 (53)	824 (44)	2209 (55)	3732 (53)	5222 (56)	4759 (46)	2447 (31)	0 (0)
Obese	111 (100)	281 (47)	658 (35)	1051 (26)	1208 (17)	832 (9)	343 (3)	0 (0)	0 (0)
Smoking status									
Never smoker	0 (0)	0 (0)	67 (4)	305 (8)	1044 (15)	2640 (28)	4586 (44)	5399 (69)	3857 (100)
Ex-smoker	0 (0)	143 (24)	508 (27)	1355 (34)	2918 (41)	4102 (44)	4159 (40)	2461 (31)	0 (0)
Current smoker	111 (100)	455 (76)	1288 (69)	2284 (58)	3141 (44)	2631 (28)	1598 (15)	0 (0)	0 (0)
Physical activity									
High	0 (0)	0 (0)	71 (4)	492 (12)	1609 (23)	2933 (31)	5376 (52)	5569 (71)	3857 (100)
Intermediate	0 (0)	78 (13)	527 (28)	1441 (36)	2671 (38)	4412 (47)	3975 (38)	2291 (29)	0 (0)
Low	111 (100)	520 (87)	1265 (68)	2091 (52)	2823 (40)	2028 (22)	992 (10)	0 (0)	0 (0)
Alcohol consumption									
Moderate	0 (0)	0 (0)	276 (15)	1553 (39)	4166 (59)	6986 (74)	8792 (85)	7199 (92)	3857 (100)
None	0 (0)	60 (10)	277 (15)	759 (19)	1127 (16)	1373 (15)	1175 (11)	661 (8)	0 (0)
Heavy	111 (100)	538 (90)	1310 (70)	1712 (43)	1810 (25)	1014 (11)	376 (4)	0 (0)	0 (0)
Socioeconomic status									
High	14 (14)	153 (27)	424 (24)	984 (25)	1829 (27)	2711 (30)	3286 (33)	2831 (37)	1494 (40)
Intermediate	44 (43)	179 (31)	635 (35)	1347 (35)	2508 (36)	3221 (35)	3644 (36)	2684 (35)	1281 (34)
Low	44 (43)	240 (42)	743 (41)	1565 (40)	2559 (37)	3197 (35)	3147 (31)	2124 (28)	960 (26)

© 2020 Nyberg ST et al. *JAMA Internal Medicine.*

	Healthy Lifestyle Score								
	0	1	2	3	4	5	6	7	8
**Women**									
Mean age (SD)	44.3 (7.9)	46.1 (9.6)	45.1 (10)	45 (10.2)	44.2 (10.6)	43.9 (10.5)	43 (10.6)	42.4 (10.6)	40.2 (11.1)
Mean BMI (SD)	33 (3)	31.4 (4.4)	29.8 (5.3)	28.5 (5.1)	26.6 (4.8)	25.1 (4.1)	23.9 (3.3)	22.9 (2.5)	21.9 (1.8)
BMI category									
Normal weight	0 (0)	0 (0)	223 (15)	838 (21)	3308 (38)	6721 (50)	11369 (66)	12866 (81)	9863 (100)
Overweight	0 (0)	130 (36)	431 (30)	1624 (41)	3292 (38)	5047 (38)	5094 (30)	3084 (19)	0 (0)
Obese	76 (100)	232 (64)	800 (55)	1499 (38)	2014 (23)	1658 (12)	742 (4)	0 (0)	0 (0)
Smoking status									
Never smoker	0 (0)	0 (0)	54 (4)	488 (12)	1846 (21)	4194 (31)	7621 (44)	9877 (62)	9863 (100)
Ex-smoker	0 (0)	65 (18)	332 (23)	1248 (31)	2785 (32)	5009 (37)	6115 (36)	6073 (38)	0 (0)
Current smoker	76 (100)	296 (82)	1065 (73)	2225 (56)	3983 (46)	4223 (31)	3469 (20)	0 (0)	0 (0)
Physical activity									
High	0 (0)	0 (0)	44 (3)	299 (8)	1468 (17)	4118 (31)	8965 (52)	11322 (71)	9863 (100)
Intermediate	0 (0)	49 (13)	316 (22)	1315 (33)	3200 (37)	5972 (44)	6076 (35)	4628 (29)	0 (0)
Low	76 (100)	313 (86)	1091 (75)	2347 (59)	3946 (46)	3336 (25)	2164 (13)	0 (0)	0 (0)
Alcohol consumption									
Moderate	0 (0)	0 (0)	339 (23)	1472 (37)	4644 (54)	8725 (65)	13421 (78)	13785 (86)	9863 (100)
None	0 (0)	117 (32)	497 (34)	1502 (38)	2647 (31)	3586 (27)	3193 (19)	2165 (14)	0 (0)
Heavy	76 (100)	245 (67)	618 (42)	987 (25)	1323 (15)	1115 (8)	591 (3)	0 (0)	0 (0)
Socioeconomic status									
High	18 (27)	61 (17)	218 (15)	674 (17)	1533 (18)	2750 (21)	4035 (24)	4433 (28)	3244 (34)
Intermediate	31 (46)	171 (48)	670 (47)	1849 (48)	4218 (50)	6709 (51)	9068 (54)	8558 (55)	5051 (52)
Low	18 (27)	120 (34)	523 (37)	1330 (34)	2632 (31)	3665 (28)	3747 (22)	2679 (17)	1378 (14)

© 2020 Nyberg ST et al. *JAMA Internal Medicine.*

eFigure 1. Meta-regression of Linear Trend Between Healthy Lifestyle Score and Disease-free Years Between Ages 40 and 75

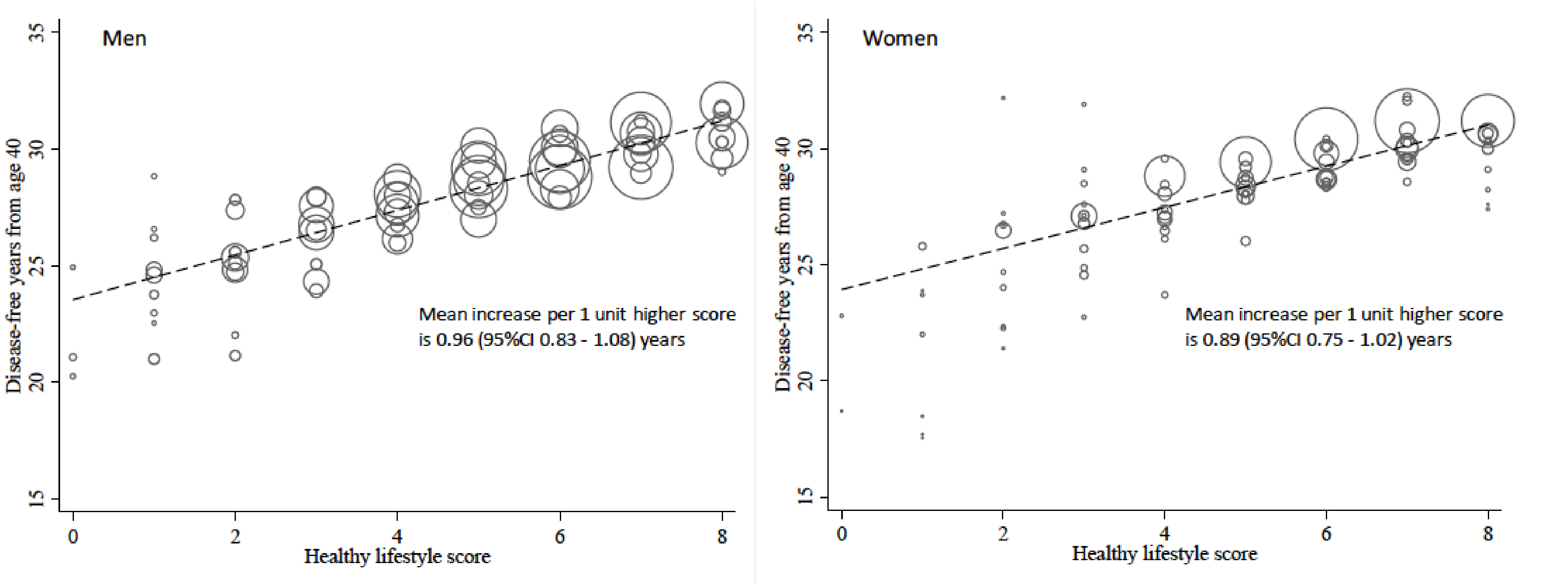

Circles represent study cohorts, with the circumference being proportional to the sample size

© 2020 Nyberg ST et al. *JAMA Internal Medicine.*

eFigure 2. Number of Disease-Free Life-Years and Age Achieved Disease-Free By Level of Healthy Lifestyle Score After Imputing Missing Predictor Values

Healthy lifestyle score		Events (total)	Mean age reached disease-free (95% CI)
Men	0	41 (107)	59.7 (56.9 — 62.5)
	1	156 (447)	64.0 (62.2 — 65.8)
	2	513 (1701)	64.8 (63.6 — 66.0)
	3	934 (3592)	66.3 (65.4 — 67.2)
	4	1464 (6416)	67.2 (66.7 — 67.7)
	5	1608 (8365)	68.7 (68.0 — 69.3)
	6	1659 (9433)	69.1 (68.5 — 69.8)
	7	1039 (7241)	69.9 (69.4 — 70.4)
	8 (lowest risk)	372 (3545)	70.6 (70.0 — 71.2)
Women	0	22 (79)	60.7 (56.6 — 64.7)
	1	61 (285)	64.3 (62.6 — 66.0)
	2	278 (1257)	65.7 (64.1 — 67.3)
	3	739 (3532)	66.6 (65.6 — 67.7)
	4	1221 (7599)	67.6 (67.1 — 68.1)
	5	1632 (11981)	68.6 (68.2 — 69.0)
	6	1681 (16314)	69.5 (68.9 — 70.1)
	7	1272 (15494)	70.3 (69.7 — 71.0)
	8 (lowest risk)	650 (9875)	70.6 (70.1 — 71.1)

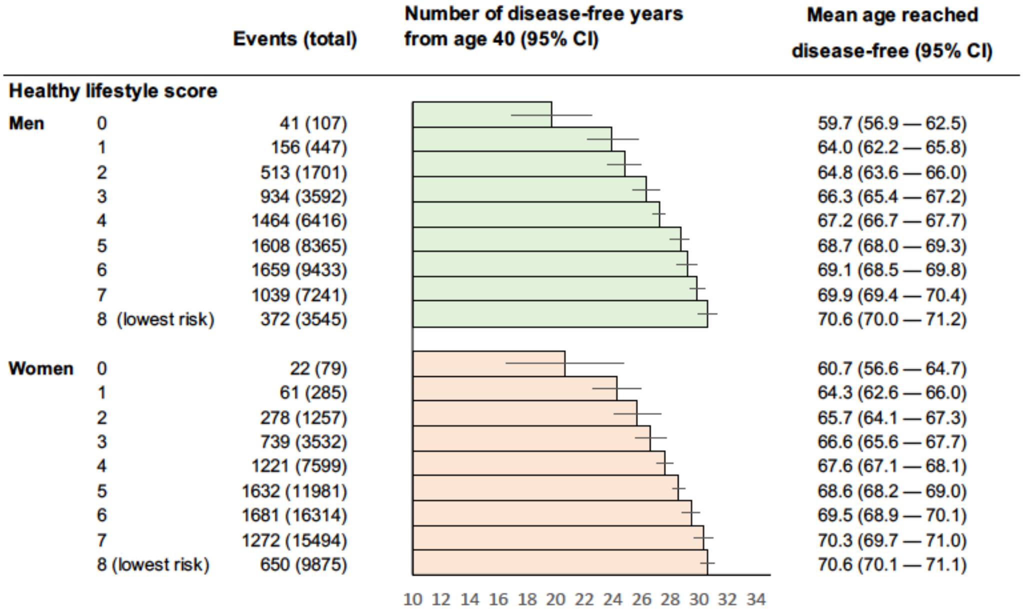

Disease-free life-years refer to the number of life-years between ages 40 and 75 that an individual was free from a diagnosis of any of the following noncommunicable diseases: type 2 diabetes, coronary heart disease, stroke, cancer, asthma, and COPD. Healthy lifestyle score included four lifestyle factors (smoking, body mass index, physical activity and alcohol consumption) which were each allocated a score based on known risk status (0, 1 or 2) and then aggregated (range 0-8).

© 2020 Nyberg ST et al. *JAMA Internal Medicine.*

eFigure 3. Number of Disease-Free Life-Years and Age Achieved Disease-Free By Level of Healthy Lifestyle Score and Socioeconomic Status

Subgroup	Healthy lifestyle score	Mean age reached disease-free (95% CI)
High SES		
	0	62·4 (57·6 — 67·2)
	1	67·2 (65·5 — 68·9)
	2	67·0 (66·0 — 68·1)
	3	68·5 (67·8 — 69·2)
	4	69·2 (68·7 — 69·7)
	5	69·8 (69·4 — 70·2)
	6	70·3 (69·9 — 70·6)
	7	70·9 (70·6 — 71·3)
	8 (lowest risk)	71·4 (71·0 — 71·7)
Intermediate SES		
	0	62·6 (59·1 — 66·1)
	1	64·6 (63·2 — 66·1)
	2	65·6 (64·8 — 66·5)
	3	66·7 (66·1 — 67·2)
	4	67·9 (67·5 — 68·4)
	5	69·3 (68·9 — 69·6)
	6	69·9 (69·6 — 70·2)
	7	70·6 (70·3 — 70·9)
	8 (lowest risk)	71·0 (70·7 — 71·4)
Low SES		
	0	62·9 (58·4 — 67·4)
	1	63·5 (61·7 — 65·2)
	2	66·1 (65·0 — 67·1)
	3	66·9 (66·2 — 67·7)
	4	67·8 (67·2 — 68·4)
	5	68·6 (68·0 — 69·1)
	6	69·6 (69·2 — 70·1)
	7	70·7 (70·2 — 71·2)
	8 (lowest risk)	70·9 (70·2 — 71·6)

Number of disease-free years from age 40 (95% CI)

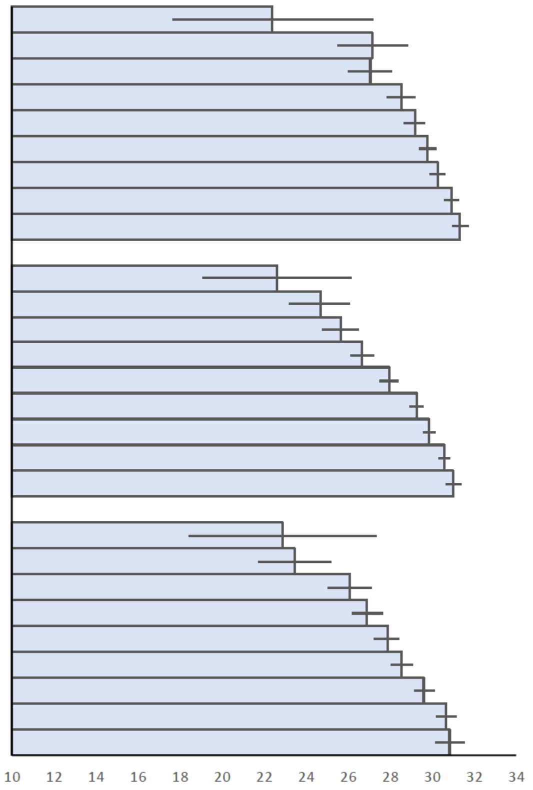

Disease-free life-years refer to the number of life-years between ages 40 and 75 that an individual was free from a diagnosis of any of the following noncommunicable diseases: type 2 diabetes, coronary heart disease, stroke, cancer, asthma, and COPD. Healthy lifestyle score included four lifestyle factors (smoking, body mass index, physical activity and alcohol consumption) which were each allocated a score based on known risk status (0, 1 or 2) and then aggregated (range 0-8). Socioeconomic status is divided into 3 levels: High, intermediate and low.

© 2020 Nyberg ST et al. *JAMA Internal Medicine.*

eFigure 4. Number of Disease-Free Life-Years and Age Achieved Disease-Free By Number of Optimal Lifestyle Factors

Optimal lifestyle factors		Events (total)	Mean age reached disease-free (95% CI)
Men	0	616 (2167)	67.2 (66.6 — 67.7)
	1	2166 (8887)	67.9 (67.5 — 68.3)
	2	2271 (12137)	69.1 (68.8 — 69.4)
	3	1234 (8769)	70.2 (69.9 — 70.5)
	4	315 (3113)	71.2 (70.8 — 71.6)
Women	0	322 (1640)	67.9 (67.3 — 68.5)
	1	1570 (9022)	68.5 (68.2 — 68.8)
	2	2381 (19051)	69.7 (69.5 — 69.9)
	3	1702 (19737)	70.8 (70.7 — 71.0)
	4	582 (8903)	71.2 (71.0 — 71.5)

Number of disease-free years from age 40 (95% CI)

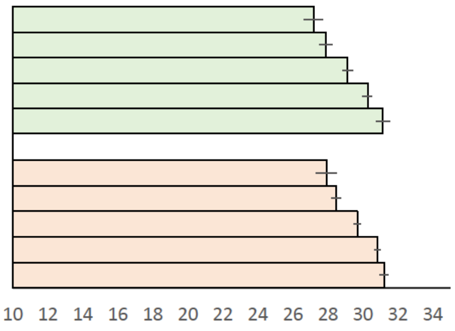

Disease-free life-years refer to the number of life-years between ages 40 and 75 that an individual was free from a diagnosis of any of the following noncommunicable diseases: type 2 diabetes, coronary heart disease, stroke, cancer, asthma, and COPD. Optimal lifestyle factors included a body-mass index <25kg/m2, never smoking, being physically active and moderate alcohol consumption.

© 2020 Nyberg ST et al. *JAMA Internal Medicine.*

eFigure 5. Number of Disease-Free Life-Years and Age Achieved Disease-Free By Number of Optimal Lifestyle Factors by Socioeconomic Status

Sex	Subgroup	Number of optimal optimal lifestyle factors	Mean age reached disease-free (95% CI)
Men	High SES	0	68.4 (67.5 — 69.2)
		1	68.7 (68.2 — 69.3)
		2	69.8 (69.4 — 70.3)
		3	70.5 (70.0 — 70.9)
		4	71.7 (71.1 — 72.2)
	Intermediate SES	0	65.9 (64.8 — 66.9)
		1	67.5 (66.8 — 68.2)
		2	68.8 (68.1 — 69.4)
		3	70.0 (69.4 — 70.6)
		4	70.8 (70.1 — 71.5)
	Low SES	0	67.3 (66.2 — 68.4)
		1	67.1 (66.4 — 67.7)
		2	68.4 (67.8 — 69.0)
		3	70.2 (69.6 — 70.8)
		4	70.1 (68.9 — 71.4)
Women	High SES	0	69.2 (68.0 — 70.4)
		1	68.9 (68.3 — 69.4)
		2	70.0 (69.6 — 70.4)
		3	71.1 (70.8 — 71.4)
		4	71.0 (70.5 — 71.4)
	Intermediate SES	0	67.9 (67.1 — 68.8)
		1	68.4 (68.0 — 68.8)
		2	69.7 (69.5 — 70.0)
		3	70.8 (70.5 — 71.0)
		4	71.3 (70.9 — 71.6)
	Low SES	0	66.9 (65.8 — 68.0)
		1	68.2 (67.7 — 68.8)
		2	69.1 (68.6 — 69.5)
		3	70.5 (70.0 — 70.9)
		4	71.5 (70.8 — 72.1)

Number of disease-free years from age 40 (95% confidence intervals)

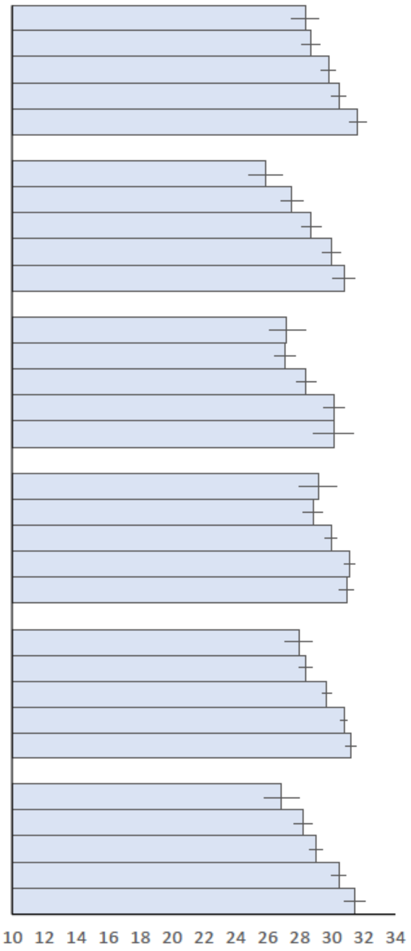

Disease-free life-years refer to the number of life-years between ages 40 and 75 that an individual was free from a diagnosis of any of the following noncommunicable diseases: type 2 diabetes, coronary heart disease, stroke, cancer, asthma, and COPD. Optimal lifestyle factors included a body-mass index <25kg/m2, never smoking, being physically active and moderate alcohol consumption. Socioeconomic status is divided into 3 levels: High, intermediate and low.

© 2020 Nyberg ST et al. *JAMA Internal Medicine.*

eFigure 6. Meta-regression of Linear Trend Between Number of Optimal Lifestyle Factors Score and Disease-free Years Between Ages 40 and 75

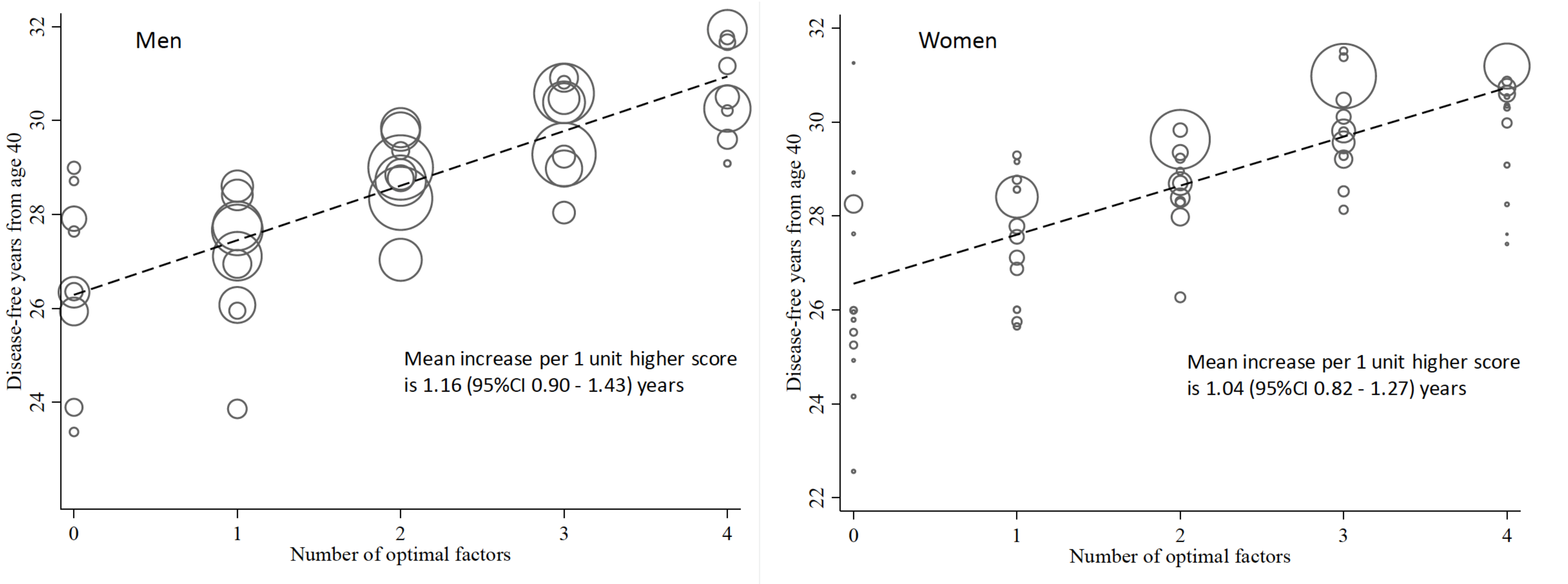

Circles represent study cohorts, with the circumference being proportional to the sample size

© 2020 Nyberg ST et al. *JAMA Internal Medicine.*

eFigure 7. Number of Disease-Free Life-Years and Age Achieved Disease-Free For 16 Lifestyle Profiles with Revised Optimal Alcohol Consumption Including Non-Drinkers and Moderate Drinkers

Number of optimal lifestyle factors		Profiles of optimal lifestyle factors: Normal weight	Never smoker	Physically active	Non or moderate drinker	Mean age reached disease-free (95% CI): Men	Women
None	Profile A	N	N	N	N	67.2 (66.6 — 67.8)	68.3 (67.4 — 69.3)
One	Profile B	Y	N	N	N	68.9 (68.2 — 69.6)	69.6 (68.7 — 70.5)
	Profile C	N	Y	N	N	68.0 (67.0 — 69.0)	67.5 (66.1 — 68.8)
	Profile D	N	N	Y	N	68.7 (68.0 — 69.5)	68.9 (67.8 — 70.0)
	Profile E	N	N	N	Y	67.5 (67.1 — 67.9)	67.8 (67.5 — 68.2)
Two	Profile F	Y	Y	N	N	69.9 (68.8 — 71.0)	71.1 (70.0 — 72.2)
	Profile G	Y	N	Y	N	69.3 (68.6 — 70.1)	70.1 (69.3 — 70.9)
	Profile H	Y	N	N	Y	69.2 (68.8 — 69.7)	70.3 (70.0 — 70.6)
	Profile I	N	Y	Y	N	70.2 (69.1 — 71.3)	68.7 (67.2 — 70.3)
	Profile J	N	Y	N	Y	69.2 (68.8 — 69.7)	68.8 (68.5 — 69.2)
	Profile K	N	N	Y	Y	68.4 (68.0 — 68.9)	69.0 (68.6 — 69.3)
Three	Profile L	Y	Y	Y	N	71.1 (70.0 — 72.1)	72.1 (71.3 — 73.0)
	Profile M	Y	Y	N	Y	70.4 (70.0 — 70.8)	71.1 (70.8 — 71.3)
	Profile N	Y	N	Y	Y	70.4 (70.0 — 70.8)	71.0 (70.8 — 71.3)
	Profile O	N	Y	Y	Y	69.5 (69.1 — 70.0)	69.7 (69.4 — 70.1)
Four	Profile P	Y	Y	Y	Y	71.2 (70.8 — 71.5)	71.2 (71.0 — 71.4)

Number of disease-free years from age 40 (95% confidence interval)

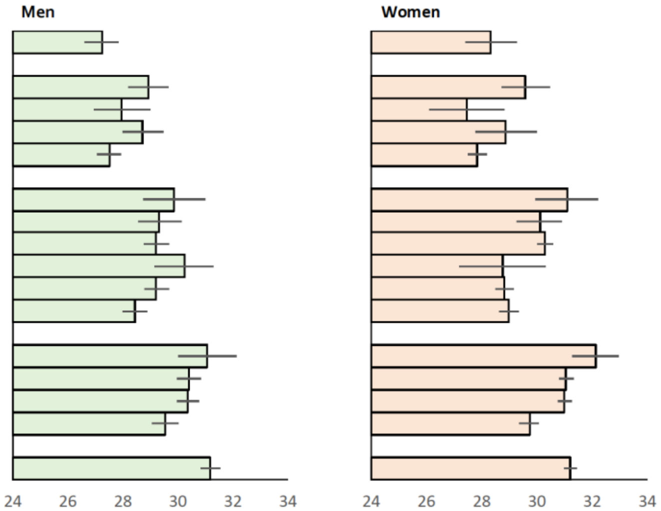

Disease-free life-years refer to the number of life-years between ages 40 and 75 that an individual was free from a diagnosis of any of the following noncommunicable diseases: type 2 diabetes, coronary heart disease, stroke, cancer, asthma, and COPD. 16 lifestyle profiles include all combinations of having 0, 1, 2, 3 or 4 of the following optimal lifestyle factors: a body-mass index <25kg/m2, never smoking, being physically active and no or moderate alcohol consumption.

© 2020 Nyberg ST et al. *JAMA Internal Medicine.*

eFigure 8. Number of Disease-Free Life-Years and Age Achieved Disease-Free By Level of Healthy Lifestyle Score After Including Two Additional Disease Endpoints (Heart Failure and Dementia)

Healthy lifestyle score		Events (total)	Mean age reached disease-free (95% CI)
Men	0	38 (93)	59.8 (55.5 — 64.1)
	1	168 (485)	63.9 (61.9 — 65.8)
	2	438 (1472)	64.7 (63.3 — 66.2)
	3	831 (3062)	65.9 (64.6 — 67.2)
	4	1261 (5435)	67.1 (66.4 — 67.8)
	5	1419 (7132)	68.5 (67.7 — 69.2)
	6	1479 (8034)	69.0 (68.3 — 69.6)
	7	925 (6236)	70.0 (69.4 — 70.6)
	8 (lowest risk)	326 (3113)	70.7 (70.1 — 71.3)
Women	0	18 (69)	60.2 (55.6 — 64.8)
	1	82 (281)	63.2 (61.5 — 65.0)
	2	268 (1138)	65.1 (63.3 — 66.9)
	3	645 (3037)	66.6 (65.5 — 67.7)
	4	1082 (6573)	67.5 (66.8 — 68.1)
	5	1445 (10453)	68.6 (68.1 — 69.0)
	6	1499 (14298)	69.5 (68.9 — 70.0)
	7	1140 (13597)	70.3 (69.7 — 70.9)
	8 (lowest risk)	599 (8903)	70.7 (70.3 — 71.1)

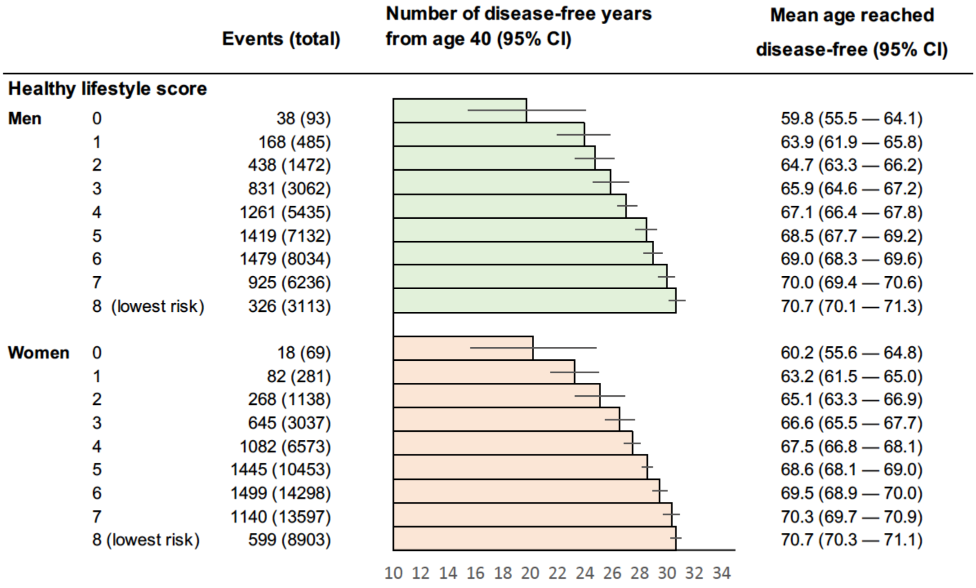

Disease-free life-years refer to the number of life-years between ages 40 and 75 that an individual was free from a diagnosis of any of the following noncommunicable diseases: type 2 diabetes, coronary heart disease, stroke, cancer, asthma, COPD, heart failure and dementia. Healthy lifestyle score included four lifestyle factors (smoking, body mass index, physical activity and alcohol consumption) which were each allocated a score based on known risk status (0, 1 or 2) and then aggregated (range 0-8).

© 2020 Nyberg ST et al. *JAMA Internal Medicine.*

eFigure 9. Number of Disease-Free Life-Years and Age Achieved Disease-Free By Number of Optimal Lifestyle Factors After Including Two Additional Disease Endpoints (Heart Failure and Dementia)

Optimal lifestyle factors		Events (total)	Number of disease-free years from age 40 (95% CI)	Mean age reached disease-free (95% CI)
Men	0	640 (2166)		66.0 (65.3 — 66.6)
	1	2244 (8884)		66.8 (66.4 — 67.2)
	2	2388 (12132)		68.2 (67.8 — 68.5)
	3	1287 (8767)		69.4 (69.1 — 69.8)
	4	326 (3113)		70.5 (70.1 — 71.0)
Women	0	343 (1639)		67.0 (66.4 — 67.7)
	1	1608 (9021)		67.9 (67.6 — 68.2)
	2	2469 (19049)		69.2 (69.0 — 69.4)
	3	1759 (19737)		70.5 (70.3 — 70.7)
	4	599 (8903)		71.0 (70.7 — 71.3)

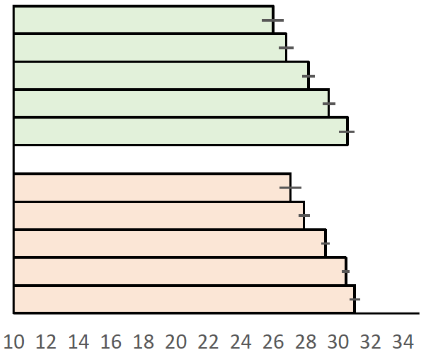

Disease-free life-years refer to the number of life-years between ages 40 and 75 that an individual was free from a diagnosis of any of the following noncommunicable diseases: type 2 diabetes, coronary heart disease, stroke, cancer, asthma, COPD, heart failure and dementia. Optimal lifestyle factors included a body-mass index <25kg/m2, never smoking, being physically active and moderate alcohol consumption.

© 2020 Nyberg ST et al. *JAMA Internal Medicine.*

eFigure 10. Number of Disease-Free Life-Years and Age Achieved Disease-Free For 16 Lifestyle Profiles After Including Two Additional Disease Endpoints (Heart Failure and Dementia)

Number of optimal lifestyle factors		Profiles of optimal lifestyle factors: Normal weight	Never smoker	Physically active	Moderate drinker	Mean age reached disease-free (95% CI): Men	Women
None	Profile A	N	N	N	N	66.0 (65.4 — 66.7)	67.1 (66.5 — 67.8)
One	Profile B	Y	N	N	N	67.5 (66.8 — 68.3)	69.8 (69.2 — 70.5)
	Profile C	N	Y	N	N	67.1 (66.2 — 68.0)	67.7 (67.1 — 68.4)
	Profile D	N	N	Y	N	67.4 (66.7 — 68.2)	68.4 (67.6 — 69.2)
	Profile E	N	N	N	Y	66.5 (66.0 — 67.0)	67.3 (66.8 — 67.7)
Two	Profile F	Y	Y	N	N	68.8 (68.0 — 69.6)	70.3 (69.8 — 70.9)
	Profile G	Y	N	Y	N	68.8 (68.0 — 69.6)	69.8 (69.2 — 70.4)
	Profile H	Y	N	N	Y	68.3 (67.7 — 68.8)	69.8 (69.5 — 70.2)
	Profile I	N	Y	Y	N	68.5 (67.5 — 69.5)	68.9 (68.1 — 69.7)
	Profile J	N	Y	N	Y	68.6 (68.0 — 69.1)	68.7 (68.2 — 69.1)
	Profile K	N	N	Y	Y	67.5 (67.0 — 68.0)	68.5 (68.1 — 69.0)
Three	Profile L	Y	Y	Y	N	70.2 (69.4 — 71.0)	70.9 (70.3 — 71.4)
	Profile M	Y	Y	N	Y	69.9 (69.3 — 70.4)	70.8 (70.5 — 71.2)
	Profile N	Y	N	Y	Y	69.6 (69.1 — 70.1)	70.8 (70.5 — 71.1)
	Profile O	N	Y	Y	Y	68.7 (68.1 — 69.2)	69.3 (68.8 — 69.8)
Four	Profile P	Y	Y	Y	Y	70.6 (70.1 — 71.0)	71.0 (70.7 — 71.3)

Number of disease-free years from age 40 (95% confidence interval)

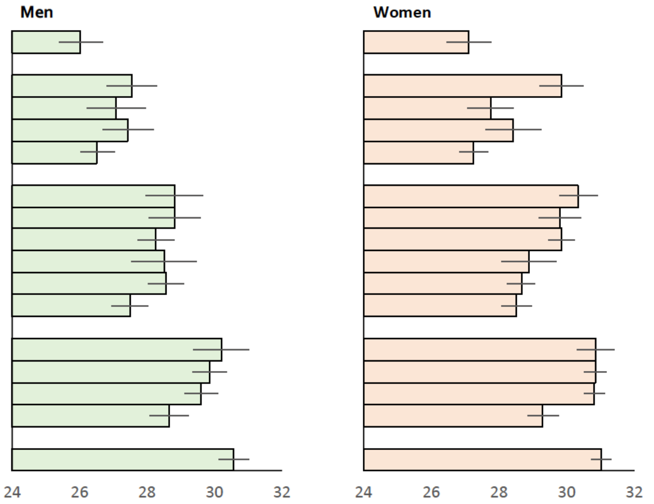

Disease-free life-years refer to the number of life-years between ages 40 and 75 that an individual was free from a diagnosis of any of the following noncommunicable diseases: type 2 diabetes, coronary heart disease, stroke, cancer, asthma, COPD, heart failure and dementia. 16 lifestyle profiles include all combinations of having 0, 1, 2, 3 or 4 of the following optimal lifestyle factors: a body-mass index <25kg/m2, never smoking, being physically active and moderate alcohol consumption.

© 2020 Nyberg ST et al. *JAMA Internal Medicine.*

eReferences

1. Pejtersen JH, Kristensen TS, Borg V, Bjorner JB. The second version of the Copenhagen Psychosocial Questionnaire. *Scand J Public Health.* 2010;38(3 Suppl):8-24.
2. Burr H, Bjorner JB, Kristensen TS, Tüchsen F, Bach E. Trends in the Danish work environment in 1990–2000 and their associations with labor-force changes. *Scand J Work Environ Health.* 2003;29(4):270-279.
3. Feveile H, Olsen O, Burr H, Bach E. Danish Work Environment Cohort Study 2005: From idea to sampling design. *Statistics in Transition.* 2007;8(3):441-458.
4. Kivimäki M, Lawlor DA, Smith GD, et al. Socioeconomic Position, Co-Occurrence of Behavior-Related Risk Factors, and Coronary Heart Disease: the Finnish Public Sector Study. *Am J Public Health.* 2007;97(5):874-879.
5. Goldberg M, Leclerc A, Bonenfant S, et al. Cohort profile: the GAZEL Cohort Study. *Int J Epidemiol.* 2007;36(1):32-39.
6. Korkeila K, Suominen S, Ahvenainen J, et al. Non-response and related factors in a nation-wide health survey. *Eur J Epidemiol.* 2001;17(11):991-999.
7. Lahelma E, Aittomaki A, Laaksonen M, et al. Cohort profile: the Helsinki Health Study. *Int J Epidemiol.* 2013;42(3):722-730.
8. Nielsen ML, Rugulies R, Christensen KB, Smith-Hansen L, Bjorner JB, Kristensen T. Impact of the psychosocial work environment on registered absence from work: a two-year longitudinal study using the IPAW cohort. *Work Stress* 2004;18(4):323-335.
9. Borritz M, Rugulies R, Bjorner JB, Villadsen E, Mikkelsen OA, Kristensen TS. Burnout among employees in human service work: design and baseline findings of the PUMA study. *Scand J Public Health.* 2006;34(1):49-58.
10. Marmot MG, Smith GD, Stansfeld S, et al. Health inequalities among British civil servants: the Whitehall II study. *Lancet.* 1991;337(8754):1387-1393.
11. Alfredsson L, Hammar N, Fransson E, et al. Job strain and major risk factors for coronary heart disease among employed males and females in a Swedish study on work, lipids and fibrinogen. *Scand J Work Environ Health.* 2002;28(4):238-248.
12. Peter R, Alfredsson L, Hammar N, Siegrist J, Theorell T, P. W. High effort, low reward, and cardiovascular risk factors in employed Swedish men and women: baseline results from the WOLF Study. *J Epidemiol Community Health.* 1998;52:540-547
13. Heikkilä K, Nyberg ST, Fransson EI, et al. Job Strain and Tobacco Smoking: An Individual-Participant Data Meta-Analysis of 166 130 Adults in 15 European Studies. PloS one. 2012;7(7):e35463.
14. Fransson EI, Heikkila K, Nyberg ST, et al. Job Strain as a Risk Factor for Leisure-Time Physical Inactivity: An Individual-Participant Meta-Analysis of Up to 170,000 Men and Women: The IPD-Work Consortium. American journal of epidemiology. 2012;176(12):1078-1089.
15. Heikkilä K, Nyberg ST, Fransson EI, et al. Job Strain and Alcohol Intake: A Collaborative Meta-Analysis of Individual-Participant Data from 140 000 Men and Women. PloS one. 2012;7(7):e40101.
16. Heikkila K, Fransson EI, Nyberg ST, et al. Job strain and health-related lifestyle: findings from an individual-participant meta-analysis of 118,000 working adults. American journal of public health. 2013;103(11):2090-2097.
17. GBD 2017 Disease and Injury Incidence and Prevalence Collaborators. Global, regional, and national incidence, prevalence, and years lived with disability for 354 diseases and injuries for 195 countries and territories, 1990-2017: a systematic analysis for the Global Burden of Disease Study 2017. *Lancet.* 2018;392(10159):1789-1858.

© 2020 Nyberg ST et al. *JAMA Internal Medicine.*

18. GBD 2017 Causes of Death Collaborators. Global, regional, and national age-sex-specific mortality for 282 causes of death in 195 countries and territories, 1980-2017: a systematic analysis for the Global Burden of Disease Study 2017. *Lancet.* 2018;392(10159):1736-1788.
19. Kivimaki M, Hamer M, Batty GD, et al. Antidepressant medication use, weight gain, and risk of type 2 diabetes: a population-based study. *Diabetes Care.* 2010;33(12):2611-2616.
20. Tabak AG, Jokela M, Akbaraly TN, Brunner EJ, Kivimaki M, Witte DR. Trajectories of glycaemia, insulin sensitivity, and insulin secretion before diagnosis of type 2 diabetes: an analysis from the Whitehall II study. *Lancet.* 2009;373(9682):2215-2221.
21. Alberti KG, Zimmet PZ. Definition, diagnosis and classification of diabetes mellitus and its complications. Part 1: diagnosis and classification of diabetes mellitus provisional report of a WHO consultation. *Diabet Med.* 1998;15(7):539-553.
22. Kivimaki M, Nyberg ST, Batty GD, et al. Job strain as a risk factor for coronary heart disease: a collaborative meta-analysis of individual participant data. *Lancet.* 2012;380(9852):1491-1497.
23. Fransson EI, Nyberg ST, Heikkila K, et al. Job strain and the risk of stroke: an individual-participant data meta-analysis. *Stroke.* 2015;46(2):557-559.
24. Heikkila K, Nyberg ST, Theorell T, et al. Work stress and risk of cancer: meta-analysis of 5700 incident cancer events in 116,000 European men and women. *BMJ* 2013;346:f165.
25. Heikkila K, Madsen IE, Nyberg ST, et al. Job strain and COPD exacerbations: an individual-participant meta-analysis. *Eur Respir J.* 2014;44(1):247-251.
26. Heikkila K, Madsen IE, Nyberg ST, et al. Job strain and the risk of severe asthma exacerbations: a meta-analysis of individual-participant data from 100 000 European men and women. *Allergy.* 2014;69(6):775-783.
27. Nyberg ST, Fransson EI, Heikkila K, et al. Job strain as a risk factor for type 2 diabetes: a pooled analysis of 124,808 men and women. *Diabetes Care.* 2014;37(8):2268-2275.

© 2020 Nyberg ST et al. *JAMA Internal Medicine.*
